# Photorespiration in eelgrass (*Zostera marina* L.): A photoprotection mechanism for survival in a CO_2_-limited world

**DOI:** 10.3389/fpls.2022.1025416

**Published:** 2022-11-11

**Authors:** Billur Celebi-Ergin, Richard C. Zimmerman, Victoria J. Hill

**Affiliations:** Department of Ocean and Earth Sciences, Old Dominion University, Norfolk, VA, United States

**Keywords:** CO_2_, non-photochemical quenching, ocean acidification, photorespiration, photosynthesis, quantum yield, seagrass

## Abstract

Photorespiration, commonly viewed as a loss in photosynthetic productivity of C3 plants, is expected to decline with increasing atmospheric CO_2_, even though photorespiration plays an important role in the oxidative stress responses. This study aimed to quantify the role of photorespiration and alternative photoprotection mechanisms in *Zostera marina* L. (eelgrass), a carbon-limited marine C3 plant, in response to ocean acidification. Plants were grown in controlled outdoor aquaria at different [CO_2_]_aq_ ranging from ~55 (ambient) to ~2121 *μ*M for 13 months and compared for differences in leaf photochemistry by simultaneous measurements of O_2_ flux and variable fluorescence. At ambient [CO_2_], photosynthesis was carbon limited and the excess photon absorption was diverted both to photorespiration and non-photochemical quenching (NPQ). The dynamic range of NPQ regulation in ambient grown plants, in response to instantaneous changes in [CO_2_]_aq_, suggested considerable tolerance for fluctuating environmental conditions. However, 60 to 80% of maximum photosynthetic capacity of ambient plants was diverted to photorespiration resulting in limited carbon fixation. The photosynthesis to respiration ratio (*P*_E_ *: R*_D_) of ambient grown plants increased 6-fold when measured under high CO_2_ because photorespiration was virtually suppressed. Plants acclimated to high CO_2_ maintained 4-fold higher *P*_E_ *: R*_D_ than ambient grown plants as a result of a 60% reduction in photorespiration. The O_2_ production efficiency per unit chlorophyll was not affected by the CO_2_ environment in which the plants were grown. Yet, CO_2_ enrichment decreased the light level to initiate NPQ activity and downregulated the biomass specific pigment content by 50% and area specific pigment content by 30%. Thus, phenotypic acclimation to ocean carbonation in eelgrass, indicating the coupling between the regulation of photosynthetic structure and metabolic carbon demands, involved the downregulation of light harvesting by the photosynthetic apparatus, a reduction in the role of photorespiration and an increase in the role of NPQ in photoprotection. The quasi-mechanistic model developed in this study permits integration of photosynthetic and morphological acclimation to ocean carbonation into seagrass productivity models, by adjusting the limits of the photosynthetic parameters based on substrate availability and physiological capacity.

## Introduction

Photosynthesis and photorespiration are competing processes due to the bi-functionality of ribulose 1,5-biphosphate carboxylase/oxygenase (Rubisco) (Spreitzer and Salvucci, 2002). Since the oxygenation reaction of Rubisco decreases photosynthetic carbon gain, it has been viewed as an inefficient legacy of evolution that might be engineered out of terrestrial plants in a quest for increased productivity (Andrews and Lorimer, 1978; Somerville, 2001; Xin et al., 2015). Recent work, however, suggests that Rubisco’s CO_2_/O_2_ specificity in different species may approach optimal acclimation to their gaseous environment in which the plants are grown (Tcherkez et al., 2006; Bathellier et al., 2018). More importantly, especially for carbon-limited seagrasses, photorespiration may serve as an important metabolic “clutch” to protect the photochemical pathway at high irradiance (Heber and Krause, 1980; Osmond, 1981; Osmond et al., 1997; Igamberdiev et al., 2001). When the Calvin Benson cycle is limited by the availability of CO_2_, continuation of light reactions over-reduces the thylakoid electron transport chain and generates O_2_ and reactive oxygen species (ROS) that potentiates oxidative stress (Voss et al., 2013). Photorespiration helps to balance the redox state and minimize the accumulation of ROS by dissipating the excess reducing equivalents (NADPH) as well as energy (ATP) (Foyer et al., 2009). By recycling the photorespired CO_2_, photorespiration may also facilitate carbon assimilation in CO_2_ limited environments, thereby minimizing photosynthetic inefficiencies resulting from C-limitation (Busch et al., 2013; Xin et al., 2015).

Photorespiration is often considered to be of minor importance in aquatic systems as a result of carbon concentrating mechanisms (CCMs) that facilitate the transport of HCO_3_^-^ and its dehydration by algal pyrenoids that effectively deliver CO_2_ to Rubisco (Frost-Christensen and Sand-Jensen, 1992; Madsen et al., 1993; Meyer et al., 2017). In today’s oceanic water (pH ~8.2), 89% of the DIC is in form of HCO_3_^-^ and only 0.5% exists as dissolved CO_2_ (Zeebe, 2012). However, not all aquatic C_3_ plants have similar efficiencies to use both forms of DIC for photosynthesis (Raven and Beardall, 2003; Raven et al., 2011; Raven and Beardall, 2014). Additionally, CO_2_ acquisition by simple diffusion through the leaf surface is more difficult for submerged plants due to the 10,000-fold lower diffusion rates of gases in a liquid environment relative to air (Borum et al., 2006). Consequently, for aquatic C_3_ plants such as seagrasses that do not use CCMs effectively, carbon limitation likely increases the photorespiratory function of Rubisco (Tolbert and Osmond, 1976; Touchette and Burkholder, 2000).

Seagrasses are flowering marine plants that evolved from terrestrial monocots in the middle Cretaceous (Larkum et al., 2006b) when higher atmospheric and oceanic CO_2_ concentrations likely supported photosynthesis and minimized photorespiration (Kuypers et al., 1999; Zeebe, 2012). In colonizing the aquatic habitat, seagrass evolved adaptations to a submerged environment that produced important anatomical differences from their terrestrial ancestors (Zimmerman et al., 1997; Larkum et al., 2006a). Seagrass leaves have no stomatal openings as gas exchange occurs across both leaf surfaces by diffusion, which uncouples carbon uptake from water relations. Seagrasses also have a lacunal system with aerenchyma extending from the roots to the leaves that facilitates the transport of O_2_ to the roots buried in permanently flooded anoxic sediments, and allows transport of CO_2_ from the roots to leaves, providing an alternative carbon source (Madsen and Sand-Jensen, 1991). Like their terrestrial ancestors, however, seagrass chloroplasts lack pyrenoids that serve as an important CCM in most aquatic algae (Meyer et al., 2017) and seagrasses are typically less efficient in utilizing HCO_3_^-^ than macroalgae (Beer et al., 1991). Although Rubisco activity in seagrasses is lower than the typical activities in freshwater emergent angiosperms and marine red algae, it is comparable to that observed in marine green and brown macroalgae (Beer et al., 1991). Simulations of nearshore seawater DIC distribution during the Cretaceous period have predicted that photosynthetic rates of seagrasses would have been similar to macroalgae (Beer and Koch, 1996). However, in today’s oceans, seagrass photosynthesis is generally considered to be carbon limited (Durako, 1993; Beer and Koch, 1996; Zimmerman et al., 1997; Invers et al., 2001).

Carbon limited photosynthesis also restricts seagrasses to shallow, high light environments, where low daytime CO_2_:O_2_ ratios in the water column may increase seagrass vulnerability to photorespiration (Buapet et al., 2013b). The photorespiratory pathway was confirmed in marine plants and macrophytes by showing that photosynthesis could be inhibited by increasing the O_2_ concentration, resulting in higher concentrations of glycolate pathway intermediates (Hough, 1974; Black et al., 1976; Burris et al., 1976; Downton et al., 1976; Hough and Wetzel, 1977; Andrews and Abel, 1979). The decreasing O_2_ evolution rates relative to electron transfer rates measured by PAM fluorometry at high irradiances in *Zostera marina* and *Halophila stipulacea* also suggested a role for photorespiration in these seagrass species (Beer et al., 1998). More recent studies demonstrated the influence of oxygen concentrations and temperature on photorespiration in seagrass that fluctuate in natural environment because of eutrophication, high community productivity and elevated ocean temperatures; and therefore, will play a role in predicting the health status of these plants in warmer climate scenarios (Buapet and Björk, 2016; Rasmusson et al., 2020). The plastochron interval, which defines leaf life span, leaf turnover and elongation rates, plays an important role in photoacclimation strategies that differ among species at the chloroplast, leaf and shoot levels (Schubert et al., 2018). However, we still do not understand how long-term acclimation to climate warming and ocean acidification/carbonation will affect photorespiration and photoprotection in seagrasses (Koch et al., 2013).

Several experiments simulating ocean acidification/carbonation impacts on time scales of hours to >1 year have quantified the positive impacts of CO_2_ availability on carbon balance, growth, survival and reproductive output in seagrasses (Zimmerman, 2021). During the most recent of these studies, the down-regulation of pigment content with increasing CO_2_ resembled the photoacclimation response to high light environment that pointed to the importance of metabolic acclimation regulating the redox state of the chloroplast in eelgrass (Zimmerman et al., 2017; Celebi-Ergin et al., 2021). Therefore, the objectives of this study were to estimate the importance of photorespiration in the marine angiosperm *Zostera marina* L. (eelgrass) under today’s oceanic carbon concentrations and explore the potential acclimation response to prolonged ocean acidification/carbonation by 1) quantifying the photochemical rates under different light and CO_2_ availability by using eelgrass grown in a high light low CO_2_ environment, i.e., representing the baseline photosynthetic capacity under today’s oceanic conditions; and 2) comparing the relative contribution of different photochemical pathways in eelgrass after 13 months of acclimation to different CO_2_ environments superimposed open daily and seasonal patterns of solar radiation, temperature and salinity.

## Materials and methods

**Table 1** provides a complete list of all abbreviations, acronyms, and symbols along with their units used throughout this paper.

**Table 1:** List of symbols, their definitions, and dimensions.

Symbol	Definition	Dimensions
Chl-*a*	Chlorophyll *a*	*µ*g cm^-2^ or mg g^-1^ FW
Chl-*b*	Chlorophyll *b*	*µ*g cm^-2^ or mg g^-1^ FW
TChl	Total Chlorophyll	*µ*g cm^-2^ or mg g^-1^ FW
TCar	Total Carotenoid	*µ*g cm^-2^ or mg g^-1^ FW
FW	Fresh Weight	mg
LA	Leaf Area	cm^2^
*A*_L_(*λ*)	Leaf absorptance	Dimensionless
*D*(*λ*)	Leaf absorbance	Dimensionless
*R*(*λ*)	Leaf reflectance	Dimensionless
*a*_L_*(*λ*)	Optical cross-section	m^2^ g^-1^ Chl-*a*
*λ*	Wavelength	nm
PAR	Photosynthetically active radiation	*µ*mol photons s^-1^ m^-2^
PUR	Photosynthetically usable radiation	*µ*mol photons s^-1^ m^-2^
*E*	Incident irradiance	*µ*mol photons s^-1^ m^-2^
*E*_k_	Photosynthesis-saturating irradiance	*µ*mol photons s^-1^ m^-2^
*P*_g_	Gross photosynthesis	*µ*mol O_2_ s^-1^ m^-2^ or *µ*mol O_2_ hr^-1^ mg^-1^ TChl
*P*_net_	Net photosynthesis	*µ*mol O_2_ hr^-1^ g^-1^ FW or *µ*mol O_2_ hr^-1^ mg^-1^ TChl
*P*_E_	light-saturated rate of gross photosynthesis	*µ*mol O_2_ s^-1^ m^-2^ or *µ*mol O_2_ hr^-1^ g^-1^ FW or *µ*mol O_2_ hr^-1^ mg^-1^ TChl
*P*_T_	True photosynthesis	
*P*_R_	Photorespiration	*µ*mol O_2_ hr^-1^ mg^-1^ TChl
*R*_D_	Dark respiration	*µ*mol O_2_ hr^-1^ g^-1^ FW
α	Photosynthetic efficiency at light-limited region of PE curve	*µ*mol O_2_ *µ*mol^-1^ photons
Φ_O2_	Quantum yield of oxygen evolution	*µ*mol O_2_ *µ*mol^-1^ photons
*F*_m_, *F*_m’_	Maximum fluorescence from dark and light adapted leaf	Dimensionless
*F*_0_, *F*_0’_	Minimum fluorescence from dark and light adapted leaf	Dimensionless
*F*_v_	Variable fluorescence	Dimensionless
Φ_PSII_	Effective Quantum yield of fluorescence ([*F*_m’_ - *F*’]/*F*_m’_)	Dimensionless
ETR	Electron transport rate	*µ*mol electrons s^-1^ m^-2^
NPQ	Nonphotochemical quenching ([*F*_m_ - *F*_m’_]/*F*_m’_)	Dimensionless

Parenthetic notation ( *λ* ) denotes wavelength dependence of the variable.

### The experimental facility and sampling from pH treatments

Eelgrass shoots used in this study were grown in an outdoor aquatic climate research facility at the Virginia Aquarium and Marine Science Center, VA, USA. The experimental design and control of manipulations for this-long term project were detailed in Zimmerman et al. (2017). Briefly, eelgrass plants, harvested in May 2013 from a subtidal population growing in South Bay, a coastal lagoon on the Virginia portion of the DelMarVa Peninsula, USA, were transplanted into 20 fiberglass open top aquaria (3 m^3^ each) plumbed with running seawater from Owl’s Creek, VA and exposed to natural sunlight. Temperature, pH, salinity, and irradiance were monitored continuously in all aquaria. Beverage-grade CO_2_ gas was used to enrich the experimental aquaria from June 2013 to October 2014 using a system of pH-controlled solenoid valves. pH treatment levels ranged from pH 6 ([CO_2(aq)_] ≅ 2121 *µ*M) to ambient (no CO_2_ addition, pH ≅ 7.7, [CO_2(aq)_] ≅ 55 *µ*M), with 0.5 pH intervals between the treatments (4 aquaria at each pH). The experimental CO_2_ manipulation produced consistently different levels of [CO_2(aq)_] and pH among the treatments day and night throughout the duration of the 13-month experiment. Plant performance was monitored monthly while environmental parameters, which varied daily and seasonally, were recorded at 10-minute intervals.

During July 2014, after 13 months of cultivation in the experimental aquaria, freshly collected 2^nd^ youngest leaves from pH 6.1 (2121 *µ*M CO_2(aq)_), pH 6.9 (371 *µ*M CO_2(aq)_) and ambient pH 7.7 (55 *µ*M CO_2(aq)_) treatments were harvested for laboratory measurements of photochemistry under fully controlled incubation conditions. Hereafter, the three treatments will be referred to as G_pH_6, G_pH_7 and G_pH_8, for simplicity. During these measurements, the daily seawater temperature in aquaria ranged from 25 to 28°C; allowing all the incubation measurements described here to be conducted at the optimal temperature of 25°C without inducing heat stress. The daily total surface irradiance ranged from 10 to 29 mol photons m^-2^ d^-1^; corresponding to more than 6 h of photosynthetically saturating irradiance (>200 *µ*mol photons m^-2^ s^-1^) per day, under which conditions the leaves of all plants should have been acclimated to a high light environment (Cummings and Zimmerman, 2003).

### Incubation measurements of leaf photochemistry

Photosynthesis and respiration rates were measured using polarographic O_2_ electrodes in temperature controlled, water-jacketed glass metabolic incubation chambers (Rank Bros., Cambridge, UK). Variable fluorescence was measured simultaneously on each leaf using a Pulse Amplitude Modulated (PAM) fluorometer (Mini PAM, Walz, Germany). Incubation seawater pH (a proxy for dissolved inorganic carbon (DIC) concentration) was measured using an epoxy mini-electrode and pH meter (Cole-Parmer) calibrated with NBS buffers. The lid of the incubation chamber was modified to hold the pH electrode in the incubation water and the miniature fiberoptic probe of the PAM device in close proximity to the leaf surface. The chamber was continuously mixed by a magnetic stirrer which homogenized the incubation medium and provided turbulent flow to reduce boundary layer limitation of gas exchange across the leaf surface. Continuous analog signals from the three sensors were recorded digitally using custom software written with LabView (2009 edition, National Instruments). Voltage data were post processed into metabolic rates using MATLAB R2014 (The MathWorks Inc.). A Kodak slide projector fitted with a halogen (ELH) bulb provided photosynthetically active radiation (PAR). The intensity of PAR was adjusted with neutral density filters and calibrated daily with a QSL 2100 scalar radiometer (Biospherical Instruments Inc.).

Concentrations of CO_2(aq)_ in the aquaria and metabolic incubation chambers were determined from measured values of pH, alkalinity, salinity and temperature using CO2SYS (Ver. 2.1; Lewis and Wallace, 1998). Leaves, harvested from plants grown at pH/CO_2_ treatments G_pH_6 ([CO_2(aq)_] = 2121 *µ*M), G_pH_7 ([CO_2(aq)_] = 371 *µ*M) and G_pH_8 ([CO_2(aq)_] =55 *µ*M), were used to measure the photosynthetic response at pH/CO_2(aq)_ levels of 6 (M_pH_6), 7 (M_pH_7) and 8 (M_pH_8). Seawater [DIC] and pH in the incubation chambers were adjusted by bubbling with a gas mixture of CO_2,_ O_2_ and N_2_ that maintained [O_2_] at air saturation (~215 *µ*M). Seawater temperature was maintained at 25°C by a circulating water bath. Leaves were cleaned of epiphytes by gently scraping with a razor blade and kept in the dark for 20 minutes before the incubation measurements. A separate three cm long pieces of leaf tissue, cut approximately one cm above the meristem, was used during each ten min dark (i.e., dark respiration) and ten min light (i.e., net photosynthesis) measurement. The pigment content and optical properties of the leaf tissues (**Table 1**) were measured after each incubation as described by Celebi-Ergin et al. (2021).

The seawater used during all incubations was collected in April 2014 from Owl’s Creek, the tidal estuary used as source water for the experimental facility (Zimmerman et al., 2017). This seawater stock, with salinity of 24 (PSS-78, (Lewis, 1980) was filtered through 0.2 *µ*m Nucleopore membrane filters and stored under refrigeration in dark bottles until used in these experiments. After incubations, alkalinity was determined on aliquots of seawater taken from the chamber using an automated potentiometric titrator (Metroohm). **Table 2** summarizes measured parameters of seawater used in metabolic incubation chamber.

**Table 2:** Distribution of dissolved inorganic carbon and dissolved oxygen concentrations in seawater during the incubation measurements of net photosynthesis at different light levels, including dark respiration measurements.

		At the start of light measurements			At the start of dark measurements		
	Target pH	Growth pH 6	Growth pH 7	Growth pH 8	Growth pH 6	Growth pH 7	Growth pH 8
Sample Size	6	7	7	5	7	7	5
	7	6	6	6	6	6	6
	8	5	5	5	5	5	5
Average pH	6	6.09 ± 0.01	6.08 ± 0.01	6.05 ± 0.01	6.09 ± 0.01	6.08 ± 0.01	6.04 ± 0.01
	7	6.91 ± 0.01	6.85 ± 0.02	6.87 ± 0.02	6.91 ± 0.01	6.84 ± 0.02	6.86 ± 0.02
	8	7.94 ± 0.05	7.95 ± 0.01	7.94 ± 0.02	8.00 ± 0.04	7.98 ± 0.01	7.98 ± 0.02
Average [TCO_2_] (µmol/L)	6	3712 ± 28	3131 ± 18	3256 ± 48	3727 ± 27	3153 ± 20	3277 ± 49
	7	2218 ± 9	1874 ± 12	1861 ± 10	2217 ± 10	1876 ± 10	1866 ± 11
	8	1857 ± 15	1534 ± 3	1535 ± 5	1837 ± 14	1526 ± 4	1526 ± 5
Average [HCO^-^_3_] (µmol/L)	6	1963 ± 0.1	1623 ± 0.0	1624 ± 0.1	1963 ± 0.1	1624 ± 0.0	1624 ± 0.1
	7	1943 ± 0.7	1610 ± 0.7	1609 ± 0.7	1943 ± 0.7	1610 ± 0.6	1609 ± 0.7
	8	1742 ± 21	1442 ± 4	1444 ± 7	1714 ± 20	1431 ± 5	1432 ± 7
Average [CO_2_] (µmol/L)	6	1748 ± 28	1506 ± 18	1631 ± 48	1762 ± 27	1528 ± 20	1652 ± 48
	7	265 ± 8	258 ± 11	246 ± 10	264 ± 10	260 ± 10	250 ± 11
	8	23 ± 3	18 ± 0.5	19 ± 0.9	19 ± 2	17 ± 0.5	17 ± 0.8
Average [O_2_] (µmol/L)	6	209.5 ± 3.1	215.0 ± 3.5	215.9 ± 2.0	212.6 ± 2.3	214.6 ± 3.0	216.0 ± 2.0
	7	214.6 ± 3.0	218.4 ± 4.3	217.5 ± 3.1	215.7 ± 3.4	219.4 ± 3.3	216.3 ± 1.7
	8	206.4 ± 2.4	215.8 ± 2.4	211.8 ± 2.7	212.2 ± 3.3	218.7 ± 1.6	215.4 ± 2.8

All measurements were conducted at 25°C using seawater with salinity of 24 ppt.

### Determination of photochemical rates

Oxygen evolution rates of each tissue were separately normalized to fresh weight, leaf area and total pigment concentration to explore the effects of phenotypic differences resulting from acclimation to different growth conditions. Parameters of photosynthesis (*P*) vs Irradiance (*E*) curves were estimated by fitting the data to a cumulative one-hit Poisson model pioneered for photosynthesis by Webb et al. (1974):


(1) $$P_{\text{net}}{\text{ = }P}_{\text{g}}{-R}_{\text{D}}$$

(2) $$P_{\text{net}}{\text{ = [ }P}_{\text{E}}\;\cdot \;\left({1\text{- e}}^{{-E\text{/ }E}_{\text{k}}}\right){\text{]-}R}_{\text{D}}$$

where *P*_net_ was the measured rate of net photosynthesis and *R*_D_ was the measured rate of dark respiration, from which the gross photosynthesis (*P*_g_) was calculated according to Equation (1). *P*_g_ was defined as a function of light, where *P*_E_ represented the light-saturated rate of gross photosynthesis that varied with [CO_2_] and [HCO_3_^-^] (sensu McPherson et al. (2015)). *E*_k_ was the irradiance threshold for photosynthetic saturation. *E* was separately defined as photosynthetically available radiation ( $\text{PAR}=\sum_{400}^{700}E[\lambda ]$ ) and as photosynthetically utilized radiation $\left(\text{PUR}=\sum_{400}^{700}\left[E\left(\lambda \right)\cdot A\left(\lambda \right)\right]\right)$ , where *A*(*λ*) was the spectral leaf absorptance that integrated the variability of light capture efficiency due to changes in leaf optical properties and pigment content/composition. The quantum yield of oxygen evolution (Φ_O2_) at different irradiances (in units of mol O_2_ mol^-1^ absorbed photon) was calculated by Φ_O2_ = *P*_g_/PUR. Maximum quantum yield was calculated as Φ_max_ = *P*_E_/*E*_k (PUR)_.

Although Equation (1) represents the typical method for determining gross photosynthesis from measured values of *P*_net_ and *R*_D_, the model does not separately account for O_2_ consumed by photorespiration in the chloroplast. It also assumes that the Mehler Ascorbate Peroxidase pathway does not affect net O_2_ exchange even though it may facilitate ATP generation and electron flow, which might be detected by fluorescence measurements (Larkum et al., 2006a). Following the principle explained by Raghavendra (2000) gross photosynthesis (*P*_g_) can be detailed as the difference between true photosynthesis (*P*_T_) and photorespiration (*P*_R_):


(3) $$P_{\text{g}}{\text{ = }P}_{\text{T}}\;{\text{– }P}_{\text{R}}$$

Under CO_2_-saturation (i.e., at low pH that increases CO_2_:O_2_ ratio in seawater, **Table 2**), *P*_R_ should approach a minimum (~ 0), so that *P*_g_ will be an approximate estimate of true photosynthetic O_2_ production (*P*_T_). In this study, O_2_ production rates measured at pH 6 were assumed to approximate the true photosynthesis (*P*_T_) for each growth condition. Therefore, photorespiration was calculated by subtracting the carbon limited *P*_g_ measured at pH > 6 from *P*_g_ measured at pH 6:


(4) $$P_{\text{R}}{\;}_{[pH>6]}=\;P_{\text{g}}{\;}_{[pH6]}-P_{\text{g}}{\;}_{[pH>6]}$$

(5) $$P_{\text{g}}{\;}_{[pH>6]}=\text{ [ }P_{\text{E}}\;\cdot \;\left({1-\text{ e}}^{{-E\text{/ }E}_{\text{k}}}\right){\text{] }}_{[pH>6]}$$

(6) $$P_{\text{g}}{\;}_{[pH6]}=\text{ [ }P_{\text{m}}\;\cdot \;\left({1-\text{ e}}^{{-E\text{/ }E}_{\text{k}}}\right){]}_{[pH6]}$$

Thus, *P*_g_ approached *P*_E_ when saturated by light and flow, and it approached the true physiological capacity (*P*_m_) when saturated by CO_2_, light and flow. In this formulation, the limit of *P*_m_ is set by availability of cellular components such as enzyme and pigment concentrations that may change under different growth conditions.

Pulsed Amplitude Modulation (PAM) fluorescence measurements were analyzed following the calculations outlined in Baker (2008). The maximum (*F*_m_) and minimum (*F*_0_) fluorescence emissions were measured in the dark after at least 10 min of acclimation while simultaneously measuring respiration. The maximum variable fluorescence yield (*F*_v_ = *F*_m_ - *F*_0_) was used to quantify the maximum quantum yield of fluorescence (*F*_v_/*F*_m_), which is a measure of maximum efficiency at which absorbed light by photosystem II (PSII) can be used for photochemistry. The maximum (*F’*_m_) and minimum fluorescence (*F*_t_) emissions induced by the short saturating pulse of PAM were measured again in the light while simultaneously measuring *P*_net_. Based on these emissions under the presence of the actinic background light, the effective quantum yield of PSII (Φ_PSII_), also known as photochemical quenching, was determined as:


(7) $$\Phi_{\text{PSII}}{=(F'}_{\text{m}}{-F}_{\text{t}}{)\;/\;F'}_{\text{m}}$$

Φ_PSII_ provides an estimate of the quantum yield of linear electron flow through PSII at a given irradiance. The other non-radiative energy loss that quenches fluorescence, called Non-Photochemical Quenching (NPQ), results from the dissipation of excess excitation energy as heat *via* the Xanthophyll cycle. NPQ was estimated as:


(8) $${\text{NPQ = ( }F}_{\text{m}}{\text{- }F\text{ '}}_{\text{m}}{\text{) / }F\text{ '}}_{\text{m}}$$

For comparisons among the treatments and incubations, NPQ and Φ_PSII_ at different light levels were fitted to a four-parameter sigmoid curve, which is commonly used for dose response analysis (Motulsky and Christopoulos, 2004), with the following formula:


(9) $${\text{NPQ = NPQ}}_{\text{min }}\text{+ }\frac{{\text{(NPQ}}_{\text{max}}{\text{+ NPQ}}_{\text{min}})}{1+{\left(\text{PUR/}EC50\right)}^{-H}}$$

where the exponent *H* was Hill slope that controlled the steepness of the dose-response curve. *EC50* was the *PUR* level required to provoke a response halfway between the baseline and maximum responses. The threshold for *NPQ_max_* was constrained to 10 based on literature values (Kalaji et al., 2014).

The electron transport rate (ETR) was estimated from Φ_PSII_ as:


(10) $${\text{ETR (}\mu \text{mol electrons m}}^{-2}{\text{s}}^{-1}\text{) = PUR }\cdot {\text{ F}}_{\text{II}}\cdot \text{Φ}{\;}_{\text{PSII}}$$

where *F*_II_ was the fraction of *PUR* captured by PSII and its light harvesting complexes (LHC). The typical value of *F*_II_ for Chlorophyta and seagrasses is about 0.5 (Figueroa et al., 2003; Larkum et al., 2006a). Photosynthetic parameters of ETR curves (i.e., ETR_max_, α_ETR_ and *E*_k -ETR_) were calculated by modifying the model of O_2_ based *P* vs *E*. curves (Equation 2):


(11) $${\text{ETR = ETR}}_{\text{max}}\;\cdot \;\left({1\text{- e}}^{{-E\text{/ }E}_{\text{k}}}\right)$$

Linear electron flow through PSII is directly related to photosynthetic oxygen production, therefore the gross photosynthesis based on fluorescence measurements (*P*_g-ETR_) were estimated from ETR as:


(12) $$P_{\text{g-ETR}}\text{ (}\mu \text{mol }{\text{O}}_{2}{\text{ m}}^{-2}{\text{s}}^{-1})=\text{ETR }\cdot \tau \;$$

where *τ* was the ratio of oxygen evolved per electron generated at PSII. Since four stable charge separations are necessary to generate one mole of O_2_ at PSII, *τ* is equal to 0.25.

### Statistical analysis

Effects of growth [CO_2_] on pigment content and optical properties of leaves were analyzed by one-way Analysis of Variance (ANOVA) followed by the Tukey multiple comparison method when significant overall effects were identified. Effects of growth [CO_2_] and measurement [CO_2_] on dark respiration rates, measured with the O_2_ evolution method, were analyzed by Analysis of Covariance (ANCOVA).

O_2_ evolution and fluorescence models were implemented by using the non-linear curve fitting tools in SigmaPlot (Systat Software Inc., Version 13.0). This tool provided the mean estimates of the model parameters with their error estimates and significances using computational procedures described by Draper and Smith (1981) and Zimmerman et al. (1987). Additionally, analysis of variance for the regression models were presented to account for the goodness of fit of the *P* vs *E* curves for each experimental condition (**Supplementary Tables 1**–**3**). Significant effects of measurement [CO_2_] and growth [CO_2_] on model parameters obtained by non-linear regression were analyzed by ANCOVA, with growth pH as the primary (categorial) factor and measurement pH as the continuous covariate.

## Results

### Photoacclimation to growth CO_2_

Pigment content and optical properties varied significantly among the leaves grown in different [CO_2_] treatments (**Table 3**). Both total chlorophyll and carotenoid content decreased with increasing growth [CO_2_], while the molar ratio of Total Car : Total Chl remained constant across CO_2_ treatments at about 0.27. The decrease in total chlorophyll resulted in an increased optical cross section (*a*_L_*(*λ*)) with growth [CO_2_], thereby reducing the package effect that results in Chlorophyll self-shading. Growth [CO_2_] increased the thickness of the unpigmented mesophyll, thereby increasing the leaf biomass per unit of surface area. These phenotypic responses, consistent with the long term acclimation responses described by Celebi-Ergin et al. (2021), had important consequences for the comparison of photosynthetic efficiencies when metabolic rates were normalized to different leaf properties.

**Table 3:** Pigment content and optical properties of leaves used in photosynthesis measurements.

Growth pH (Growth [CO_2_])	pH8 (55 *µ*M)	pH7 (371 *µ*M)	pH6 (2121 *µ*M)
Sample Size (n)	16	18	18
FW per LA (mg cm^-2^)	25.8 ± 1.33 ^a^	27.1 ± 0.92 ^a^	36.0 ± 1.52 ^b^
Total Chl per LA (µg Chl cm^-2^)	31.2 ± 1.22 ^a^	27.0 ± 1.20 ^b^	20.8 ± 0.86 ^c^
Total Chl per FW (mg Chl g^-1^ FW)	1.25 ± 0.07 ^a^	1.01 ± 0.05 ^b^	0.59 ± 0.03 ^c^
Total Car per LA (µg Car cm^-2^)	8.16 ± 0.28 ^a^	7.25 ± 0.25 ^b^	5.61 ± 0.17 ^c^
Chl a:b	3.44 ± 0.04 ^a^	3.73 ± 0.07 ^b^	3.61 ± 0.04 ^a,b^
TCar:TChl	0.26 ± 0.00 ^a^	0.27 ± 0.00 ^a^	0.27 ± 0.00 ^a^
Absorptance at 550nm	0.38 ± 0.01 ^a^	0.37 ± 0.01 ^a^	0.29 ± 0.01 ^b^
Absorptance at 680nm	0.75 ± 0.01 ^a,b^	0.75 ± 0.01 ^a^	0.73 ± 0.01 ^b^
*a*_L_*(680) (m^2^ g^-1^ Chl)	5.90 ± 0.33 ^a^	6.73 ± 0.29 ^a^	8.10 ± 0.24 ^b^

Effects of growth pH on mean concentrations ( ± 1 SE) were analyzed by one-way ANOVA. Different letters represent significant differences among the growth pH for each parameter compared by Tukey method at *p*< 0.05. FW, Fresh Weight; LA, Leaf Area; Chl, Chlorophyll; Car, Carotenoid.

### Light response curves of oxygen flux

Rates of dark respiration, whether normalized to biomass (*R*_D (FW)_) or leaf area (*R*_D (LA)_), were not affected by growth [CO_2_] or instantaneous variations of [CO_2_] within the metabolic incubation chambers (**Table 4**). Therefore, the average rate of dark respiration for all samples combined was 5.96 ± 0.31 *µ*mol O_2_ hr^-1^ g^-1^ FW or 0.50 ± 0.03 *µ*mol O_2_ m^-2^ s^-1^. Dark respiration rates were also independent of pH within the range examined here, indicating no negative impact of changing ionic composition on respiration.

**Table 4:** Dark respiration (R_D_) rates measured with O_2_ evolution method and estimated by non-linear model fit to *P vs E* curves.

Growth pH	Measurement pH	Measured Dark Respiration Averages (*µ*mol O_2_ hr^-1^ g^-1^ FW)				Modeled Dark Respiration (*µ*mol O_2_ hr^-1^ g^-1^ FW)		Modeled Dark Respiration (*µ*mol O_2_ s^-1^ m^-2^)	
6	6	4.61 ± 0.75				4.73 ± 1.34		0.45 ± 0.10	
	7	5.31 ± 0.62				5.50 ± 1.15		0.51 ± 0.08	
	8	5.84 ± 0.47				5.90 ± 0.63		0.69 ± 0.07	
7	6	6.40 ± 1.14				6.90 ± 3.06		0.51 ± 0.12	
	7	6.73 ± 1.20				7.08 ± 3.02		0.53 ± 0.22	
	8	5.03 ± 0.91				5.04 ± 1.88		0.37 ± 0.12	
8	6	6.52 ± 0.93				6.83 ± 2.06		0.46 ± 0.07	
	7	6.18 ± 0.75				6.71 ± 2.19		0.46 ± 0.14	
	8	7.29 ± 1.26				7.29 ± 1.05		0.56 ± 0.10	
ANCOVA of Measured R_D_		*df*	SS	MS	F		*p*	
Growth pH		2	11.551	5.776	1.173		0.318	
Measurement pH		1	0.531	0.531	0.108		0.744	
Growth pH x Measurement pH		2	10.206	5.103	1.037		0.363	
Residual		46	226.435	4.922	–		–	
Total		51	255.682	5.013	–		–	

Rates are normalized both to Fresh Weight (FW) and Leaf Area. Effects of measurement pH and growth pH on measured R_D_ were analyzed by ANCOVA. Sample size for each condition is given in **Table 2**.

**Figure 1:**
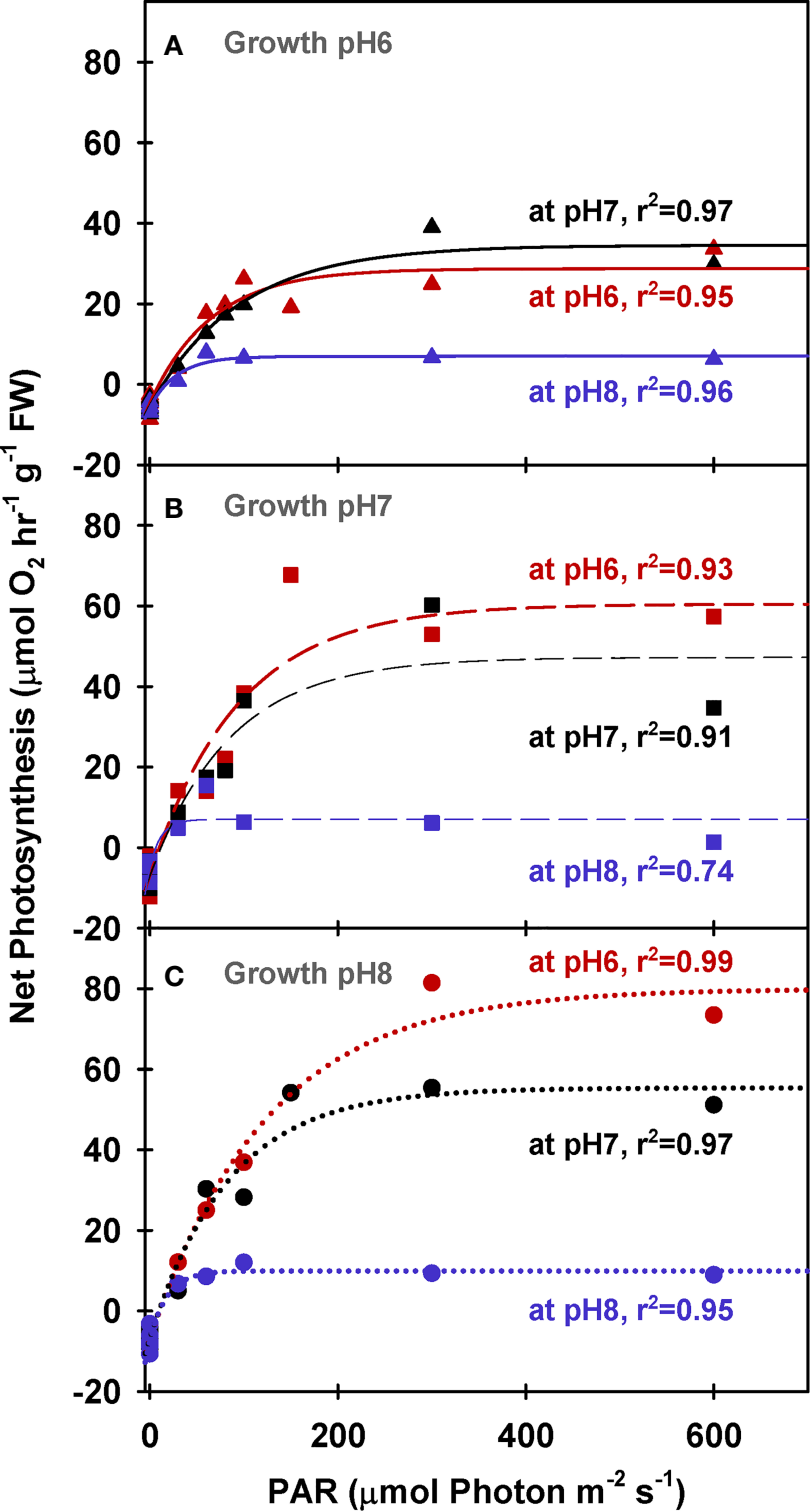
Net photosynthesis of eelgrass leaves (biomass normalized) as a function of irradiance. O_2_ production rates were measured at different pH levels (red: pH6, black: pH7 and blue: pH8) using leaves grown at pH6 (2121 *µ*M CO_2(aq)_) **(A)**, pH7 (371 *µ*M CO_2(aq)_) **(B)** and ambient pH8 (55 *µ*M CO_2(aq)_) **(C)**. Curves were fit using Equation (2).

In contrast, net O_2_ production rates increased with light and incubation [CO_2_] for all plants, regardless of the CO_2_ environment in which they were grown (**Figure 1**). The biomass specific rate of light-saturated photosynthesis (*P*_E (FW)_) averaged 14.1 *µ*mol O_2_ hr^-1^ g^-1^ FW at low incubation [CO_2_] for all plants and increased as a function of incubation [CO_2_] (**Figure 1**). However, *P*_E (FW)_ of the plants grown under ambient conditions (G_pH_8) was twice as sensitive to increasing incubation [CO_2_] as plants grown under the highest CO_2_ enrichment (G_pH_6) (**Table 5**, 86.8 vs 33.5 *µ*mol O_2_ hr^-1^ g^-1^ FW at M_pH_ 6 respectively). This difference was associated with 2-fold higher biomass specific pigment content of the plants grown under ambient [CO_2_] (**Table 3**). Thus, low rates of oxygen evolution by ambient plants in their natural low CO_2_ environment resulted mainly from photorespiration and not the lack of photosynthetic capacity characterized by light harvesting, electron transport and carbon fixation.

**Table 5:** Model estimates (mean ± 1 SE) of photosynthesis parameters generated by non-linear regression fit to the experimental data using Equation (2) (N.S. stands for non-significant parameter estimate).

Model Estimates		Measurement pH		
	Growth pH	6.0	7.0	8.0
*P*_E_ (*µ*mol O_2_ hr^-1^ mg^-1^ Chl) G_pH_ x M_pH_: p=0.570 G_pH_: p=0.583 M_pH_: p=0.002	6	70.2 ± 4.3	55.2 ± 3.7	24.5 ± 2.1
	7	68.0 ± 3.2	49.3 ± 3.1	12.5 ± 3.1
	8	62.6 ± 2.4	44.9 ± 4.0	20.3 ± 2.4
*P*_E_ (*µ*mol O_2_ hr^-1^ g^-1^ FW) G_pH_ x M_pH_: p=0.240 G_pH_: p=0.185 M_pH_: p=0.014	6	33.5 ± 2.8	40.0 ± 2.5	12.9 ± 1.1
	7	67.4 ± 7.1	54.3 ± 6.5	12.1 ± 2.9
	8	86.8 ± 4.7	62.1 ± 4.6	17.2 ± 1.7
*P*_E_ (*µ*mol O_2_ s^-1^ m^-2^) G_pH_ x M_pH_: p=0.233 G_pH_: p=0.199 M_pH_: p=0.007	6	3.6 ± 0.2	3.5 ± 0.2	1.5 ± 0.1
	7	5.8 ± 0.3	4.3 ± 0.5	0.9 ± 0.2
	8	5.8 ± 0.2	4.3 ± 0.3	1.5 ± 0.2
ETR_max_ (*µ*mol Electron s^-1^ m^-2^) G_pH_ x M_pH_: p=0.573 G_pH_: p=0.482 M_pH_: p=0.119	6	35.3 ± 0.4	41.0 ± 3.3	22.4 ± 0.5
	7	93.1 ± 2.6	68.3 ± 4.2	32.4 ± 1.1
	8	58.4 ± 5.8	82.2 ± 5.5	22.8 ± 0.7
α_ETR_ (*µ*mol Electron *µ*mol^-1^ absorbed Photon) G_pH_ x M_pH_: p=0.696 G_pH_: p=0.735 M_pH_: p=0.257	6	0.45 ± 0.01	0.50 ± 0.07	0.52 ± 0.03
	7	0.42 ± 0.01	0.48 ± 0.04	0.44 ± 0.04
	8	0.50 ± 0.08	0.46 ± 0.03	0.52 ± 0.04
Φ_max_ (*µ*mol O_2_ *µ*mol^-1^ absorbed Photon) G_pH_ x M_pH_: p=0.263 G_pH_: p=0.314 M_pH_: p=0.100	6	0.077 ± 0.01	0.084 ± 0.01	0.107 ± 0.03
	7	0.079 ± 0.01	0.079 ± 0.02	0.14 ± 0.24
	8	0.083 ± 0.01	0.074 ± 0.01	0.081 ± 0.03
*E*_k_ (*µ*mol absorbed photon s^-1^ m^-2^) from ‘PG per Chl vs PUR’ G_pH_ x M_pH_: p=0.391 G_pH_: p=0.348 M_pH_: p=0.006	6	47.5 ± 7.0	36.4 ± 6.0	14.5 ± 4.9
	7	64.2 ± 7.4	43.9 ± 6.9	4.7 ± 15.8
	8	68.6 ± 7.2	57.1 ± 13.3	17.4 ± 7.1
*E*_k_ (*µ*mol photon s^-1^ m^-2^) from ‘PG per FW vs PAR’ G_pH_ x M_pH_: p=0.523 G_pH_: p=0.501 M_pH_: p=0.046	6	65.0 ± 14.5	94.7 ± 14.8	28.4 ± 8.6
	7	94.0 ± 24.2	85.3 ± 25.1	11.6 ± 23.5
	8	124.9 ± 18.6	83.9 ± 16.9	18.3 ± 8.7
*E*_k_ (*µ*mol absorbed photon s^-1^ m^-2^) from ‘ETR vs PUR’ G_pH_ x M_pH_: p=0.560 G_pH_: p=0.469 M_pH_: p=0.117	6	78.5 ± 2.0	82.2 ± 15.6	43.1 ± 2.9
	7	220.1 ± 10.3	142.6 ± 19.0	72.8 ± 7.3
	8	117.5 ± 27.8	180.1 ± 22.9	44.2 ± 3.8

Significant effects of measurement pH (M_pH_) and growth pH (G_pH_) on mean estimates were analyzed by ANCOVA.

For all plants, increased incubation [CO_2_] also increased the irradiance required to saturate photosynthetic oxygen production (*E*_k (PAR)_ and *E*_k (PUR)_); rather than changing the photosynthetic efficiency (*α*) within the light limited region of *P* versus *E* response curves (**Table 5** and **Supplementary Table 1**). Overall, photoacclimation of eelgrass leaves to ocean carbonation increased *E*_k (PUR)_ values from 17 to 44 and 48 *µ*mol absorbed photon s^-1^ m^-2^ for pH 8 (55 *µ*M CO_2(aq)_), pH 7 (371 *µ*M CO_2(aq)_) and pH 6 (2121 *µ*M CO_2(aq)_) respectively.

**Figure 2:**
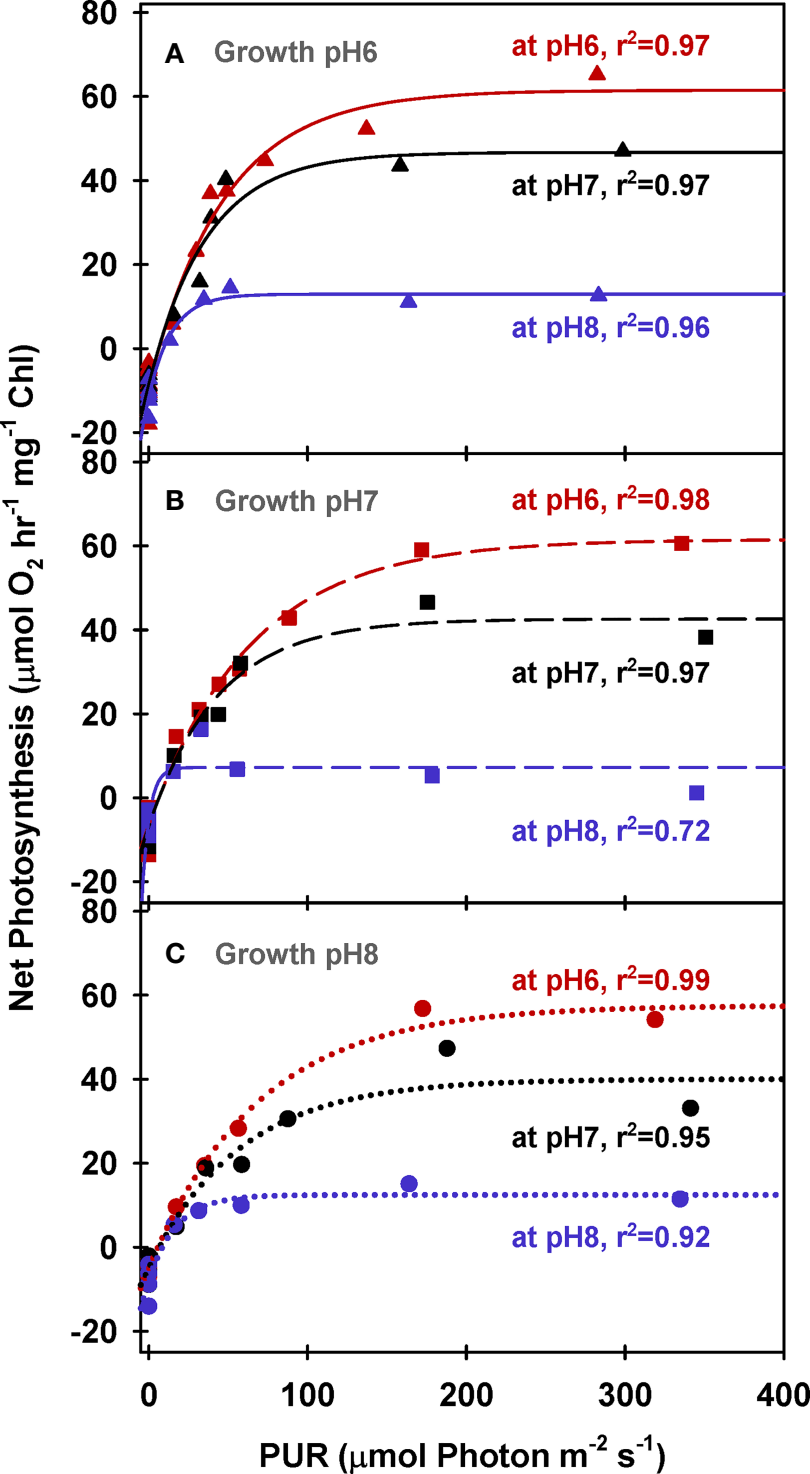
Net photosynthesis of eelgrass leaves (Chlorophyll normalized) as a function of absorbed irradiance. O_2_ production rates were measured at different pH levels (red: pH6, black: pH7 and blue: pH8) using leaves grown at pH6 (2121 *µ*M CO_2(aq)_) **(A)**, pH7 (371 *µ*M CO_2(aq)_) **(B)** and ambient pH8 (55 *µ*M CO_2(aq)_) **(C)**. Curves were fit using Equation (2).

**Figure 3:**
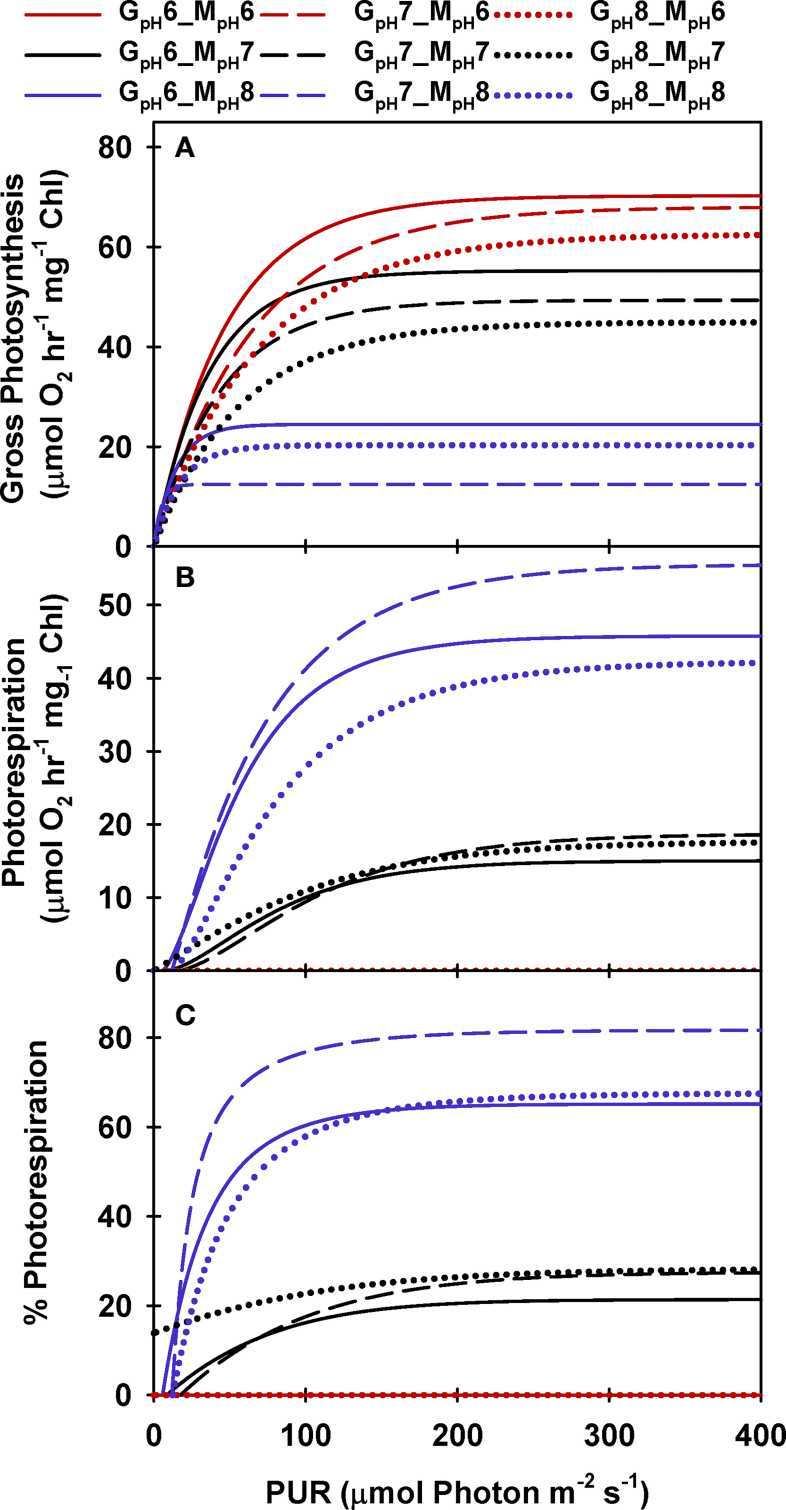
Modeled gross photosynthesis **(A)** and photorespiration **(B, C)** of eelgrass leaves as a function of absorbed irradiance. Colors represent different pH/CO_2_ levels at which the measurements (M_pH_) were performed; line styles represent the different pH/CO_2_ levels at which the plants were grown (G_pH_). Photorespiration at M_pH6_ were zero.

Chlorophyll specific rates of light-saturated photosynthesis (*P*_E (Chl)_) were the same for all plants grown at different CO_2_ environments and produced an identical response to incubation [CO_2_] (**Figure 2**). Consequently, the O_2_ production efficiency per unit chlorophyll was not affected by the CO_2_ environment in which the plants were grown (**Table 5** and **Supplementary Table 2**) and the stimulatory effect of [CO_2_] on O_2_ evolution was instantaneous (**Figure 3A**). The most likely explanation for this instantaneous response would be a reversible and light dependent O_2_ consuming process involving the chloroplast, such as photorespiration (*P*_R_), that is competitively inhibited by increasing [CO_2_]. Therefore, for all plants grown under all treatments, *P_E (Chl)_* rates at high incubation [CO_2_] (i.e., at M_pH_6) were assumed to be the true physiological photosynthetic capacity (*P*_m_) under light, carbon and flow saturation. Based on this assumption, photorespiration rates were quantified by solving the Equation 4 with the chlorophyll specific gross photosynthesis models (**Figure 3B**). Normalizing the models to pigment, rather than biomass or area, eliminated the effect of morphological differences among the plants on net oxygen metabolism.

Like photosynthesis, photorespiration increased with light under constant [CO_2_], but decreased with increasing incubation [CO_2_], as carboxylation became increasingly favored over oxygenation (**Figure 3B**). Predicted *P*_R_ rates increased rapidly with light to a maximum of 60 to 80% of *P*_m_ at low [CO_2_] (i.e., M_pH_8) (**Figure 3C**). When aqueous [CO_2_] was equal to aqueous [O_2_] (at M_pH_7, **Table 2**), maximum *P*_R_ rates were only 20% of *P*_m_, which is equivalent to the inherent carboxylation: oxygenation ratio of Rubisco.

**Figure 4:**
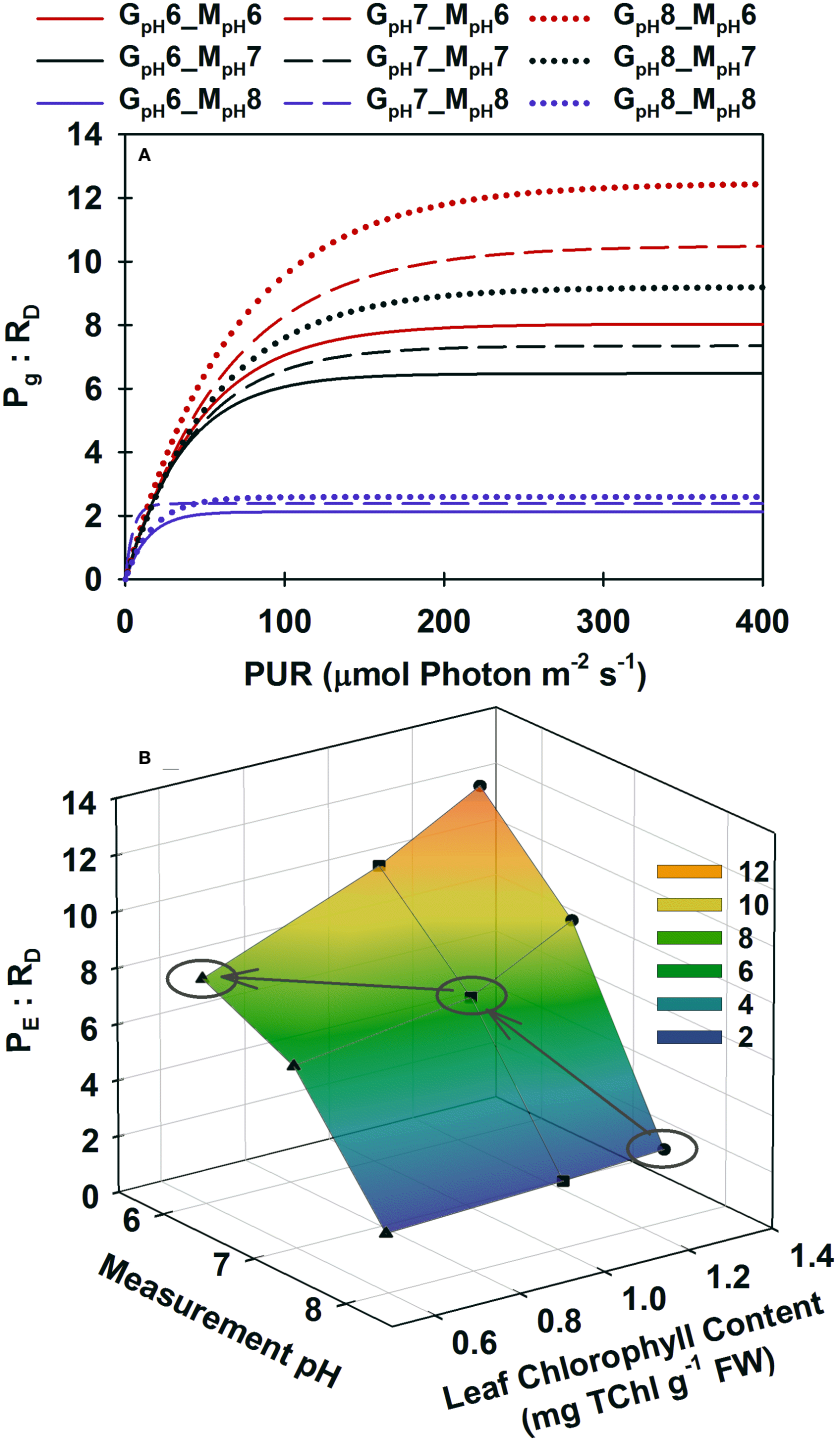
Modeled ratio of gross photosynthesis to dark respiration as a function of absorbed irradiance **(A)** and as a function of Chlorophyll content at saturating irradiances **(B)**. Colors represent different pH/CO_2_ levels at which the measurements (M_pH_) were performed; line styles and symbols (▲, at pH6 ◼ at pH7, ● at ambient pH) represent the different pH/CO_2_ levels at which the plants were grown (G_pH_). **(B)** Ellipses highlight when plants from different treatments were incubated at their corresponding growth pH/CO_2_. Gray arrows show the trajectory of *P*_E_ *: R*_D_ as a result of phenotypic acclimation to the increasing CO_2_ environment.

All plants reached the lowest gross photosynthesis to dark respiration ratio (*P*_g_ *: R*_D_) of 2 at low incubation [CO_2_] when light saturated (**Figure 4A**). This ratio increased instantaneously when saturated with CO_2_ in the incubation medium, maximally up to 12 for ambient plants (G_pH_8). However, the *P*_E_ *: R*_D_ ratio of high CO_2_ grown plants peaked at 8 when saturated with CO_2_ in the incubation medium, illustrating the consequence of pigment acclimation on metabolic balance of plants grown in a high CO_2_ environment (**Figure 4B**, grey arrows). Having excess pigment content in a CO_2_-limited environment (as observed in ambient plants) did not improve the *P*_E_ *: R*_D_ under normal growth conditions even though it allowed the instantaneous 6-fold increase of *P*_E_ *: R*_D_ when incubation [CO_2_] increased. High CO_2_ acclimated plants, on the other hand, maintained a 4-fold higher *P*_E_ *: R*_D_ above ambient plants at their respective growth [CO_2_] even though pigment content of the high CO_2_ plants decreased by half.

### Light response curves of variable fluorescence

**Figure 5:**
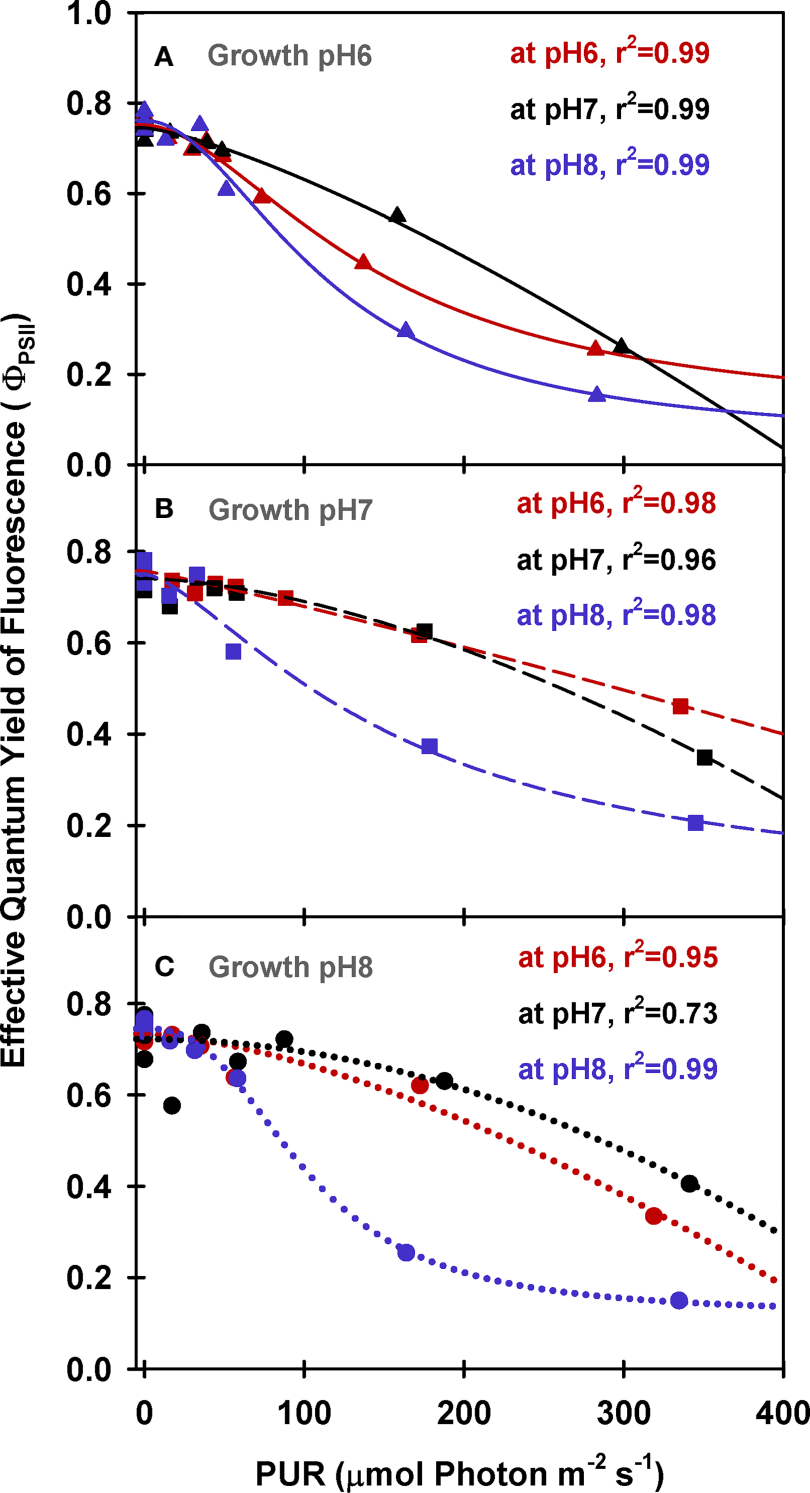
PAM fluorescence parameters of eelgrass leaves as a function of absorbed irradiance. PAM fluorescence measurements were performed at different pH/CO_2_ levels (red: pH6, black: pH7, blue: pH8) using leaves grown at pH6 (2121 *µ*M CO_2(aq)_) **(A)**, pH7 (371 *µ*M CO_2(aq)_) **(B)** and ambient pH8 (55 *µ*M CO_2(aq)_) **(C)**. Curves were fit using Equation (9).

**Figure 6:**
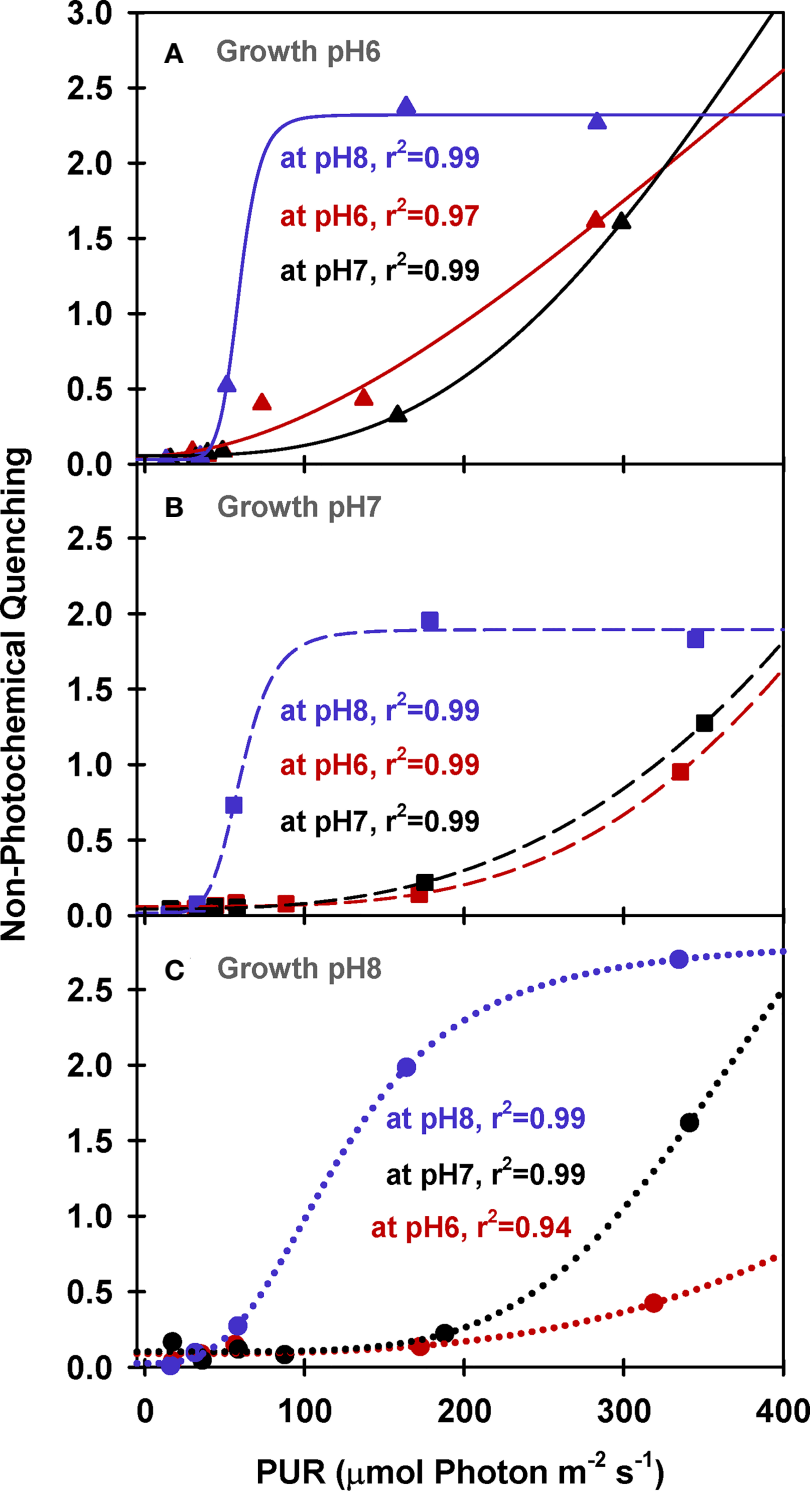
PAM fluorescence parameters of eelgrass leaves as a function of absorbed irradiance. PAM fluorescence measurements were performed at different pH/CO_2_ levels (red: pH6, black: pH7, blue: pH8) using leaves grown at pH6 (2121 *µ*M CO_2(aq)_) **(A)**, pH7 (371 *µ*M CO_2(aq)_) **(B)** and ambient pH8 (55 *µ*M CO_2(aq)_) **(C)**. Curves were fit using Equation (9).

Maximum quantum yields of fluorescence by dark-adapted leaves were above 0.7 regardless of incubation [CO_2_], indicating leaves from all growth treatments were healthy during the experiments (Φ_PSII_ at PUR 0 *µ*mol absorbed photon s^-1^ m^-2^, **Figure 5**). For all plants, effective quantum yields of fluorescence (Φ_PSII_) decreased faster with increasing light when the incubation [CO_2_] was low (M_pH_8). The decreased photochemical yield resulted from rapid induction of non-photochemical quenching (NPQ) when [CO_2_] was limited under light saturation (**Figure 6**). Increasing the growth [CO_2_], however, caused the light-dependent onset of NPQ to increase, as the NPQ pathway saturated more quickly due the decreased carotenoid content of leaves grown under high [CO_2_] (**Table 3**).

Under saturating irradiance (350 *µ*mol absorbed photon s^-1^ m^-2^, **Figure 6**), NPQ values of ambient plants increased 5-fold as incubation [CO_2_] became increasingly limiting. In contrast, the plants grown under high [CO_2_] (G_pH_6) yielded the same light-saturated NPQ of 2.5 regardless of incubation [CO_2_]. The dynamic range of NPQ regulation in ambient grown plants in response to instantaneous changes in [CO_2_] suggests considerable tolerance for fluctuating environmental conditions (**Figure 6C**).

**Figure 7:**
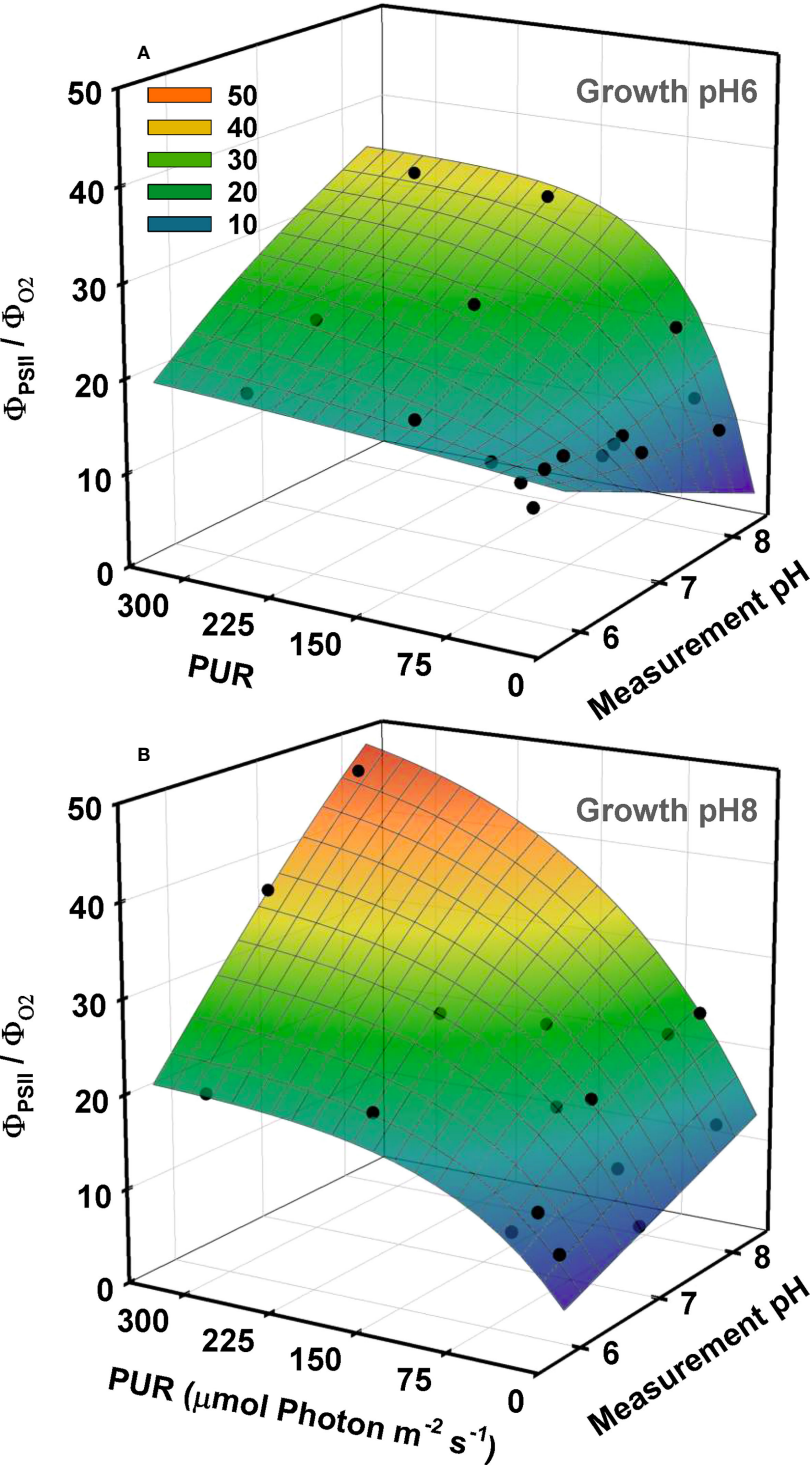
Ratio of the quantum yield of fluorescence (Ф_PSII_) to the quantum yield of oxygen (Ф_O2_) as a function of light and incubation pH/CO_2_. O_2_ production and fluorescence were measured simultaneously at different pH levels using eelgrass leaves grown at different CO_2_ treatments. Yields were calculated using PUR. Growth specific 3D relationships, at pH6 (2121 *µ*M CO_2(aq)_) **(A)** and ambient pH8 (55 *µ*M CO_2(aq)_) **(B)**, were generated by the combination of non-linear and linear regression fits. First, the Ф_PSII_/Ф_O2_ ratio as a function of PUR were described by the exponential rise to maximum models separately for each incubation pH/CO_2_. Parameter estimates of these non-linear regression models were fitted as a function of incubation pH using linear regression.

The relation between quantum yield of fluorescence (Φ_PSII_) and quantum yield of oxygen evolution (Φ_O2_) was nonlinear, and their ratios were closest to the theoretical value of 8 only at low light and high [CO_2_] conditions (**Figure 7**). For this ratio to be higher than 8, either less than half of the photons are directed to PSII (i.e., F_II_<0.5, Equation 10), and/or more than four electrons are processed to evolve one mole of oxygen (i.e., *τ*<0.25, Equation 12). Both outcomes highlight deviation from linear electron flow. For ambient plants, Φ_O2_ decreased faster than Φ_PSII_ with increasing light resulting in a drastic increase in Φ_PSII_:Φ_O2_, especially at their growth CO_2_ (G_pH_8), suggesting that these plants were using an alternative pathway to maintain electron flow without the production or consumption of O_2_.

**Figure 8:**
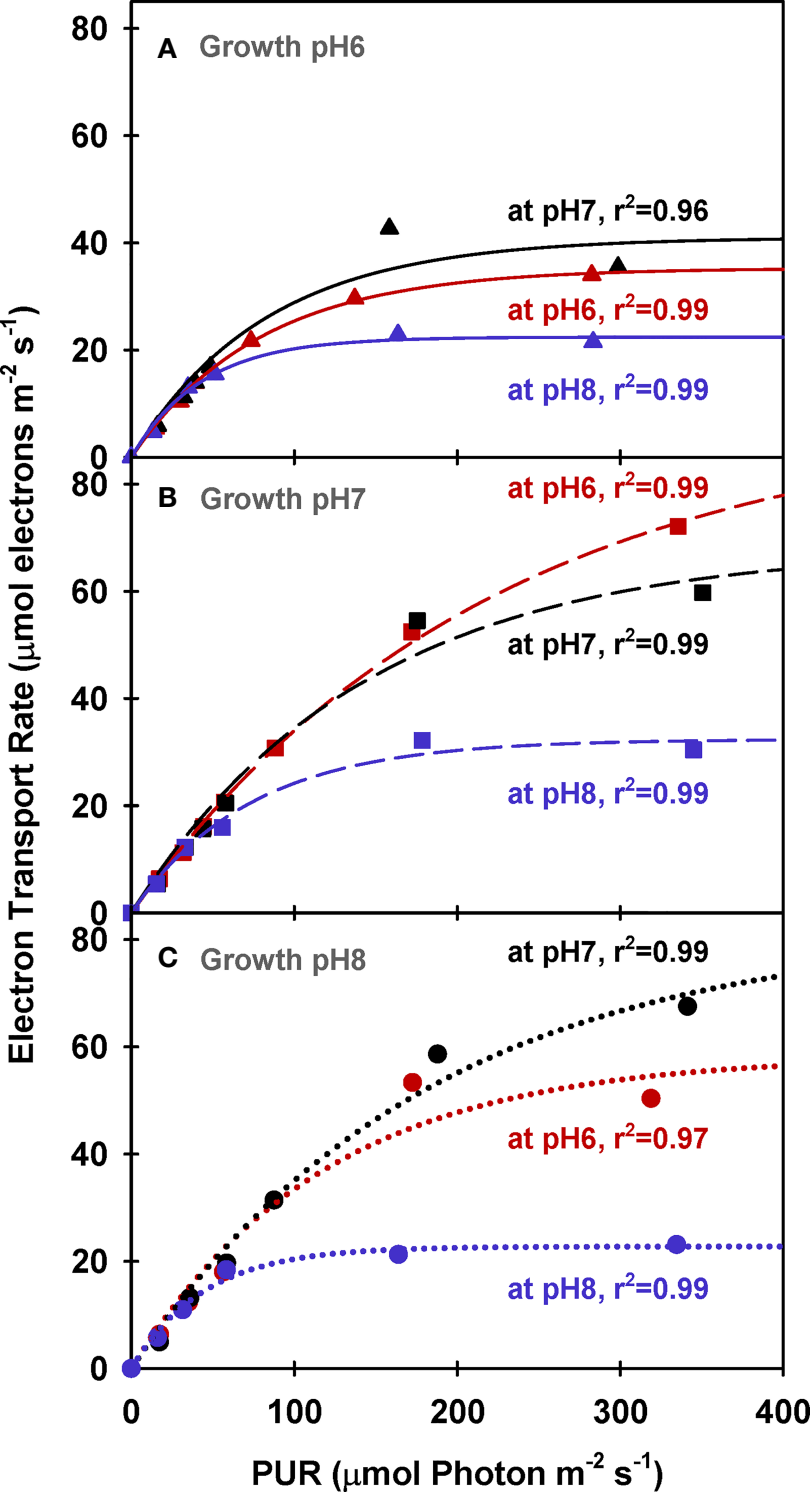
Electron transport rates of eelgrass leaves as a function of absorbed irradiance. PAM measurements were performed at different pH/CO_2_ levels (red: pH6, black: pH7 and blue: pH8) using leaves grown at pH6 (2121 *µ*M CO_2(aq)_) **(A)**, pH7 (371 *µ*M CO_2(aq)_) **(B)** and ambient pH8 (55 *µ*M CO_2(aq)_) **(C)**. Curves were fit using Equation (11).

**Figure 9:**
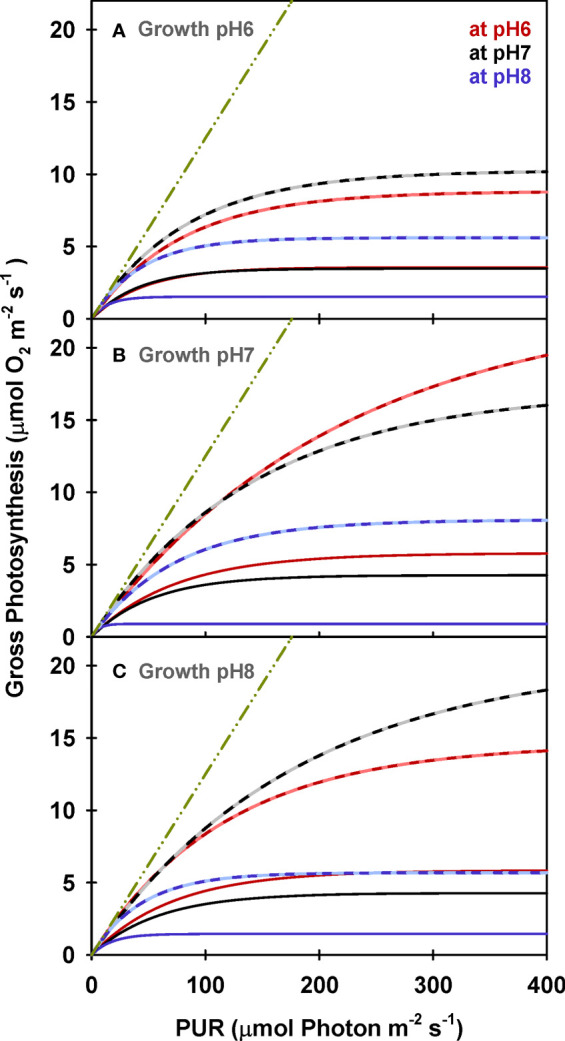
Modeled gross photosynthesis of eelgrass leaves as a function of absorbed irradiance for plants growing at pH6 (2121 *μ*M CO_2(aq)_) **(A)**, pH7 (371 *μ*M CO_2(aq)_) **(B)** and ambient pH8 (55 *μ*M CO_2(aq)_) **(C)**. Solid lines are calculated from leaf area normalized O_2_ production rates (Equation 1) and dashed lines are estimated from ETR measurements (Equation 12). Colors represent incubation pH/CO_2_ levels. Green dot-dashed lines represent the theoretical O_2_ production per absorbed photon under non-limiting environmental conditions.

Similar to net photosynthesis rates, electron transport rates (ETR) of all plants increased with light and were lowest at low incubation [CO_2_] (i.e., M_pH_8) (**Figure 8** and **Table 5**). However, the increase of ETR_max_ with incubation CO_2_ was not consistent among the plants due to the non-monotonic response of Φ_PSII_ with incubation [CO_2_] (**Figure 5**), in agreement with the findings by Celebi (2016). Only ETR_max_ of plants grown at G_pH_7 increased consistently with increasing incubation [CO_2_]. For all incubation experiments, PUR levels required to saturate ETR (*E*_k-ETR_) were consistently higher than the *E*_k_ values required to saturate O_2_ production (**Table 5** and **Supplementary Table 3**). For all plants, estimated gross photosynthesis based on ETR were also higher than the gross photosynthesis measured by the O_2_ evolution method (**Figure 9**). However, this overestimation was not consistent among plants grown at different CO_2_ environments. The *P*_E (LA)_ to ETR_max_ ratio was around 0.1 for pH 6 and pH 8 plants when incubated at pH 6 and pH 8, instead of the theoretical value (*τ*) of 0.25 (**Table 5**).

## Discussion

Long-term growth under high [CO_2_] produced a remarkable combination of morphological and metabolic changes in eelgrass. Although pigment content decreased in plants grown at high CO_2_, leaf biomass increased as a direct result of the CO_2_-stimulated increase in photosynthetic carbon gain. The equivalent responses of chlorophyll normalized O_2_ production rates to increased incubation [CO_2_], independent of the growth CO_2_, allowed us to quantify the impact of [CO_2_] on photorespiration in eelgrass because the instantaneous difference in O_2_ production rates in CO_2_-saturated vs. CO_2_-limited incubation media corresponded to the amount of O_2_ consumed in the photorespiratory pathway. Thus, photosynthesis and photorespiration as a function of light for each growth condition were precisely predictable using the *P* versus *E* curves, although the responses to incubation CO_2_ differed between biomass and pigment normalization due to changes in leaf morphology. Presently, models of eelgrass performance do not consider these long-term morphological and metabolic acclimation responses (Zimmerman, 2003; Zimmerman, 2006; Zimmerman et al., 2015). Thus, the quasi-mechanistic model developed in this study permits integration of the photosynthetic and morphological acclimation due to ocean carbonation into seagrass productivity models, by adjusting the limits of the photosynthetic parameters based on substrate availability and physiological capacity.

Morphological acclimation and regulation of pigment content, Rubisco activity, light capture and carbon fixation as a function of CO_2_ availability have been previously observed in freshwater angiosperms (Madsen et al., 1996). Increasing *P*_g_ *: R*_D_ due to the enhancing impact of [CO_2_] on *P*_E_ was detected even in short term (2-6 weeks) studies using temperate and tropical seagrass species without any CO_2_ effect on pigment content (Zimmerman et al., 1997; Ow et al., 2015). Long term studies, moreover, reported significant increases in total shoot biomass, carbon allocation to roots and rhizomes (blue carbon), shoot survival and reproductive output by eelgrass in response to CO_2_ availability (Palacios and Zimmerman, 2007; Zimmerman et al., 2017). Despite the decreases in pigment content and leaf absorptance observed here, plants grown at high CO_2_ were able to maintain higher *P*_g_ *: R*_D_ ratios than plants grown under ambient CO_2_; indicating a strong coupling between the regulation of photosynthetic structure and metabolic carbon demands. This coupling between photosynthetic regulation and growth might be poor for organisms that undergo photodamage because photosynthesis might accommodate the biochemical costs associated with protection and recovery rather than fueling the energy towards growth (Barra et al., 2014). On the other hand, the eelgrass used in these experiments show no sign of photodamage, either in the growth aquaria or in laboratory incubations even when photosynthesis was carbon limited but light saturated.

When measured at low [CO_2_], plants grown under ambient CO_2_ had the same photosynthetic O_2_ production as the plants grown at high [CO_2_]. These same photosynthetic rates highlighted the apparent lack of carbon concentrating mechanisms inducible by low CO_2_ availability in eelgrass, in contrast with marine algae and cyanobacteria that are capable of upregulating their carbon concentrating mechanisms *via* e.g., generation of pyrenoids, carboxysomes and periplasmic carbonic anhydrases when CO_2_ availability becomes limiting (Björk et al., 1993; Raghavendra, 2000; Falkowski and Raven, 2007; Meyer et al., 2017). This was also consistent with the limited sensitivity of eelgrass photosynthesis to the aqueous presence of acetazolamide, an inhibitor of periplasmic carbonic anhydrase (McPherson et al. (2015) and Celebi-Ergin - unpublished data). Seagrasses living in shallow estuarine environments, like the Chesapeake Bay eelgrass used in this study, are subject to highly variable CO_2_/pH levels daily and seasonally, which might explain the unresponsiveness of CCMs for ambient plants (Buapet et al., 2013a; Duarte et al., 2013; Ruesink et al., 2015; Zimmerman et al., 2017; Cyronak et al., 2018). Similarly, all plants had the same *P*_E (Chl)_ when measured at saturating [CO_2_] due to minimized *P*_R_, indicating all plants approached the same physiological oxygen production capacity per available photosynthetic apparatus (i.e., *P*_m (Chl)_ was constant across all treatments). Therefore, the difference in *P*_E_ *: R*_D_ among growth [CO_2_] treatments when all were incubated at high [CO_2_] resulted from the downregulation of light harvesting components by plants grown in the high CO_2_ environment.

Despite phenotypic acclimation across the CO_2_ gradient, the maximum photosynthetic efficiency (Φ_max_) remained constant for all plants (~0.08 mol O_2_ mol^-1^ absorbed photon) but photosynthesis-saturating light levels (*E*_k_) increased, as was predicted by the model of McPherson et al. (2015). Photosynthetic efficiency within and among seagrass species vary with efficiency of light absorption and the subsequent conversion of that energy into carbon assimilation (Ralph et al., 2007). Although the observed values of *α* in this study were in agreement with previous estimates for eelgrass (Frost-Christensen and Sand-Jensen, 1992), constant *α* across different CO_2_ regimes represents an interesting contrast to that observed in terrestrial C3 plants in which *α* increases with [CO_2_] availability (Raghavendra, 2000). This difference between responses of aquatic and terrestrial plants may result because CO_2_ responses are coupled to water stress in terrestrial plants but not in aquatic plants. The increased *E*_k_ and *P*_E_ values for high CO_2_ acclimated plants will decrease the estimates of *H*_sat_ (i.e., average daily period of *P*_E_) required to maintain positive carbon balance for the whole plant. The *H*_sat_ requirement is a useful modeling tool in predicting the depth distribution of eelgrass in variable light environments (Dennison, 1987; Zimmerman et al., 1991; Zimmerman et al., 1994; Zimmerman et al., 1995).

A strong correlation between diurnal NPQ cycle (i.e., xanthophyll cycle) and high light exposure has been confirmed for eelgrass to avoid photodamage under fluctuating light environments (Ralph et al., 2002). High light acclimated eelgrass leaves have higher NPQ activity, and higher photosynthetic capacity, than low light acclimated leaves (Ralph and Gademann, 2005). Here, we demonstrated a similar effect of [CO_2_] availability on NPQ activity. Under ambient CO_2_ concentrations, the onset of photosynthesis CO_2_ limitation (*E*_k_) occurred at lower irradiance, accelerating the diversion of excess photon absorption to NPQ, likely using the xanthophyll cycle as a photoprotective mechanism to prevent photoinhibition. The high CO_2_ incubations reduced this carbon limitation and increased the *E*_k_, consequently reducing NPQ. Due to increased *E*_k_, the same light environment became less damaging at high CO_2_, which may explain the reduction in both photosynthetic and photoprotective pigments observed in response to growth CO_2_. Thus, by reducing CO_2_ limitation of Rubisco, ocean carbonation should also reduce the vulnerability of eelgrass to excess reactive oxygen species (ROS) and therefore the need for photoprotection.

The simultaneous measurements of variable fluorescence, and O_2_ flux performed here yielded quantitative estimates of changes in photoprotective pathways of eelgrass acclimated to different CO_2_ environments. The difference between the theoretical O_2_ evolution (i.e., the linear increase of O_2_ with light) and the ETR estimates of gross photosynthesis (*P*_g-ETR_) was most pronounced for plants grown at high CO_2_, accounting for the absorbed photons that did not contribute to the electron transport pathway (not exciting electrons at PSII), but explained by quenching pathways, such as fluorescence and NPQ. This trend was consistent with their lower area-specific O_2_ production rates at high CO_2_ incubations when compared to pH7 and ambient pH grown plants. These plants downregulated their pigment content but increased the light-dependent NPQ at lower irradiances even at high incubation [CO_2_]. This may indicate that phenotypic acclimation to ocean carbonation by downregulating the photosynthetic apparatus reduces the role of photorespiration but increases the role of NPQ in photoprotection.

On the other hand, as observed in all treatments, the difference between the ETR estimated gross photosynthesis (*P*_g-ETR_) and the gross photosynthesis measured by oxygen production (*P*_g_) may result from inaccurate assumptions of F_ii_ and/or *τ* (Equation 12). In theory, 8 photons absorbed equivalently both by PSI and PSII (F_ii_= 0.5) excites a total of 4 electrons producing 1 mole of O_2_ (*τ* =0.25). This equilibrium of linear electron flow is valid when there is no limitation of resources such as CO_2_ and/or accumulation of byproducts such as reducing equivalents and ROS (Scheibe et al., 2005; Dietz and Pfannschmidt, 2011; Pfannschmidt and Yang, 2012). Under limiting conditions, this balance shifts towards pathways that ensure the optimal redox state of the chloroplast resulting in altered photon: electron: O_2_ ratios (Foyer et al., 2012).

Following the linear assumption that 4 electrons produce 1 O_2_ (*τ* = 0.25) resulted in overestimation of the PG_ETR_ in all treatments. Since the molecular chemistry of water splitting at PSII is well-known, *τ* can only be reduced in an apparent sense. This apparent ratio can result from the excitation of four electrons (as detected with PAM) either without producing O_2_, indicating cyclic electron flow around PSII, or consumption of O_2_ in the chloroplast that would remain undetected by the gas exchange method (Foyer et al., 2009; Ananyev et al., 2017). Two possible pathways to explain a reduction in *τ* due to O_2_ consumption are (i) the Mehler reaction and (ii) photorespiration. The Mehler reaction increases the pH gradient that may induce NPQ (Demmig-Adams and Adams, 1996; Kanazawa and Kramer, 2002). However, in this study NPQ induction did not happen until Φ_PSII_ values fell below 0.6 while O_2_ yield continuously decreased. Therefore, the observed nonlinearity between quantum yield of fluorescence and quantum yield of oxygen most likely resulted from O_2_ consumption *via* photorespiration, which probably represents the primary pathway to remove excess O_2_ buildup and use the ATP energy from light reactions for this purpose. NPQ was then triggered when photorespiration is incapable of consuming enough ATP to lower the pH gradient forming across lumen at very high irradiances.

Other pathways that keep the electron flow continuous without contributing to CO_2_ assimilation are the malate valve and the cyclic electron flow around PSI, which triggers NPQ by generating a pH gradient (Munekage et al., 2004; Johnson, 2005; Miyake, 2010). If PSI cyclic electron flow plays an important role, then the assumption of half of the absorbed photons going to PSII (e.g., F_ii_=0.5) would be inaccurate. Although PAM measurements are easily made under field conditions (including underwater) and provide non-intrusive information about the photoprotection of eelgrass through NPQ, the fluorescence measurements with PAM overestimate *P*_g-ETR_ and therefore are not equivalent to true carbon assimilation. Fluorescence measurements may account for the number of absorbed photons used in electron excitation but not necessarily towards the rates of oxygen production/consumption or carbon assimilation, especially when alternative electron sinks are available (Beer et al., 1998). Still, by having quantified the ratio of Φ_PSII_ to Φ_O2_ as a function of light and carbon availability in response to acclimation to ocean carbonation, the alternative electron pathways can be accounted for in the future estimation of photosynthesis in eelgrass.

To conclude, photorespiration likely provides an important metabolic clutch to protect the photochemical pathway in CO_2_-limited eelgrass by maintaining electron flow to prevent the inhibitory damage to photosystems due to light saturation when carbon assimilation is limited by CO_2_ supply. In addition to providing a photoprotective role, photorespiration could serve multiple purposes by connecting different metabolic pathways that allow instantaneous energy and reductant modulation under fluctuating environmental conditions. Further, photorespiration may provide a carbon concentrating mechanism *via* recycling of photorespired CO_2_ and removing excess intracellular O_2_. Therefore, even though carbon limitation causes eelgrass photosynthesis to saturate at relatively low light levels in the modern ocean, longer daily periods of saturating irradiances might be required to keep the photosynthetic apparatus running to produce ATP to support photorespiration. Consequently, understanding photoprotection mechanisms employed by these remarkable plants that are permanently rooted in highly variable shallow-water environments, becomes important when high water column productivity causes [O_2_] to rise and [CO_2_] to fall just as daily irradiances begin to peak. More importantly, this study demonstrated that acclimation of photoprotective mechanisms in response to CO_2_ availability accounted for the previously reported physiological acclimations of enhanced growth and survival of this species under ocean acidification scenarios.

## Data availability statement

The original contributions presented in the study are included in the article/**Supplementary Material**. Further inquiries can be directed to the corresponding author.

## Author contributions

BC-E, RZ and VH conceived the project. BC-E, RZ and VH performed the research. BC-E and RZ analyzed the data. BC-E, RZ and VH wrote the article. All authors contributed to the article and approved the submitted version.

## Funding

Financial support for this research was provided by the National Science Foundation (Award OCE-1061823 to RZ and VH), Virginia Sea Grant/NOAA (Award NA14OAR4170093 to RZ and BC-E) and the Department of Ocean, Earth & Atmospheric Sciences, Old Dominion University (to BC-E). This research was performed in partial completion of the requirements for the Ph.D. degree (Oceanography) at Old Dominion University.

## Acknowledgments

Thanks to W. M. Swingle and the staff of the Virginia Aquarium & Marine Science Center for assistance with maintenance of the experimental facility, and to D. Ruble, M. Jinuntuya, C. Zayas-Santiago, T. Cedeno and M. Smith for assistance with experimental procedures and maintenance of the experimental plants.

## Conflict of interest

The authors declare that the research was conducted in the absence of any commercial or financial relationships that could be construed as a potential conflict of interest.

## Publisher’s note

All claims expressed in this article are solely those of the authors and do not necessarily represent those of their affiliated organizations, or those of the publisher, the editors and the reviewers. Any product that may be evaluated in this article, or claim that may be made by its manufacturer, is not guaranteed or endorsed by the publisher.
