# Supplementary material for: Photorespiration in eelgrass (Zostera marina L.): A photoprotection mechanism for survival in a CO2-limited world
Source: Front Plant Sci. 2022 Nov 11;13:1025416. doi: 10.3389/fpls.2022.1025416 (PMC9692008; doi:10.3389/fpls.2022.1025416)
Supplement: Supplementary file 1 [file DataSheet_1.pdf]

Table S1. Results of non-linear regression analysis for biomass specific photosynthesis versus E_{PAR} curves. P_E: Light saturated Gross Photosynthesis (μmol O_2 hr^{-1} g^{-1} Fresh weight), $E_{k\ (PAR)}$: photosynthesis-saturating irradiance calculated using photosynthetically active irradiance (μmol photon s^{-1} m^{-2}), R_D: Dark respiration (μmol O_2 hr^{-1} g^{-1} Fresh weight).

Model parameters								Analysis of Variance (Corrected for the mean of the observations)					
Growth pH	Measurement pH		Coefficient	SE	t	*p*	r^2		DF	SS	MS	F	*p*
6	6.0	P_E	33.5	2.8	12.1	<0.0001	0.950	Regression	2.0	2642.4	1321.2	103.6	<0.0001
		$E_{k\ (PAR)}$	65.0	14.5	4.5	0.001		Residual	11.0	140.3	12.8		
		R_D	4.7	1.3	3.5	0.005		Total	13.0	2782.7	214.1		
	7.0	P_E	40.0	2.5	15.8	<0.0001	0.973	Regression	2.0	2690.4	1345.2	163.7	<0.0001
		$E_{k\ (PAR)}$	94.7	14.8	6.4	0.000		Residual	9.0	74.0	8.2		
		R_D	5.5	1.2	4.8	0.001		Total	11.0	2764.4	251.3		
	8.0	P_E	12.9	1.1	12.0	<0.0001	0.961	Regression	2.0	350.9	175.5	87.0	<0.0001
		$E_{k\ (PAR)}$	28.4	8.6	3.3	0.013		Residual	7.0	14.1	2.0		
		R_D	5.9	0.6	9.3	<0.0001		Total	9.0	365.0	40.6		
7	6.0	P_E	67.4	7.1	9.5	<0.0001	0.925	Regression	2.0	9127.2	4563.6	67.4	<0.0001
		$E_{k\ (PAR)}$	94.0	24.2	3.9	0.003		Residual	11.0	744.6	67.7		
		R_D	6.9	3.1	2.3	0.045		Total	13.0	9871.8	759.4		

Table S1. *continued*

Growth pH	Measurement pH		Coefficient	SE	t	*p*	r^2		DF	SS	MS	F	*p*
7	7.0	P_E	54.3	6.5	8.4	<0.0001	0.911	Regression	2.0	5168.3	2584.1	45.9	<0.0001
		$E_{k\ (PAR)}$	85.3	25.1	3.4	0.008		Residual	9.0	506.9	56.3		
		R_D	7.1	3.0	2.3	0.044		Total	11.0	5675.2	515.9		
	8.0	P_E	12.1	2.9	4.2	0.004	0.741	Regression	2.0	352.1	176.0	10.0	0.009
		$E_{k\ (PAR)}$	11.6	23.5	0.5	0.637		Residual	7.0	123.1	17.6		
		R_D	5.0	1.9	2.7	0.031		Total	9.0	475.2	52.8		
8	6.0	P_E	86.8	4.7	18.5	<0.0001	0.985	Regression	2.0	10376.8	5188.4	231.2	<0.0001
		$E_{k\ (PAR)}$	124.9	18.6	6.7	0.000		Residual	7.0	157.1	22.4		
		R_D	6.8	2.1	3.3	0.013		Total	9.0	10533.9	1170.4		
	7.0	P_E	62.1	4.6	13.5	<0.0001	0.965	Regression	2.0	7411.8	3705.9	124.5	<0.0001
		$E_{k\ (PAR)}$	83.9	16.9	5.0	0.001		Residual	9.0	267.9	29.8		
		R_D	6.7	2.2	3.1	0.014		Total	11.0	7679.8	698.2		
	8.0	P_E	17.2	1.7	10.3	<0.0001	0.947	Regression	2.0	681.0	340.5	62.1	<0.0001
		$E_{k\ (PAR)}$	18.3	8.7	2.1	0.073		Residual	7.0	38.4	5.5		
		R_D	7.3	1.0	7.0	0.000		Total	9.0	719.3	79.9		

Table S2. Results of non-linear regression analysis for pigment specific photosynthesis versus E_{PUR} curves. P_E: Light saturated Gross Photosynthesis (μmol O_2 hr^{-1} mg^{-1} Chlorophyll), $E_{k\ (PUR)}$: photosynthesis-saturating irradiance calculated using photosynthetically usable irradiance (μmol absorbed photon s^{-1} m^{-2}), R_D: Dark respiration (μmol O_2 hr^{-1} mg^{-1} Chlorophyll).

Model parameters								Analysis of Variance (Corrected for the mean of the observations)					
Growth pH	Measurement pH		Coefficient	SE	t	*p*	r^2		DF	SS	MS	F	*p*
6	6.0	P_E	70.2	4.3	16.4	<0.0001	0.974	Regression	2.0	9701.4	4850.7	209.2	<0.0001
		$E_{k\ (PUR)}$	47.5	7.0	6.8	<0.0001		Residual	11.0	255.0	23.2		
		R_D	8.7	1.8	4.9	0.001		Total	13.0	9956.4	765.9		
	7.0	P_E	55.2	3.7	15.0	<0.0001	0.970	Regression	2.0	5681.2	2840.6	146.3	<0.0001
		$E_{k\ (PUR)}$	36.4	6.0	6.1	0.000		Residual	9.0	174.7	19.4		
		R_D	8.5	1.8	4.8	0.001		Total	11.0	5855.9	532.4		
	8.0	P_E	24.5	2.1	11.4	<0.0001	0.956	Regression	2.0	1269.6	634.8	76.1	<0.0001
		$E_{k\ (PUR)}$	14.5	4.9	3.0	0.020		Residual	7.0	58.4	8.3		
		R_D	11.5	1.3	8.9	<0.0001		Total	9.0	1327.9	147.5		
7	6.0	P_E	68.0	3.2	21.0	<0.0001	0.984	Regression	2.0	8533.8	4266.9	343.0	<0.0001
		$E_{k\ (PUR)}$	64.2	7.4	8.7	<0.0001		Residual	11.0	136.8	12.4		
		R_D	6.5	1.3	5.0	0.000		Total	13.0	8670.7	667.0		

Table S2. *continued*

Growth pH	Measurement pH		Coefficient	SE	t	*p*	r^2		DF	SS	MS	F	*p*
7	7.0	P_E	49.3	3.1	15.9	<0.0001	0.973	Regression	2.0	4417.7	2208.9	162.8	<0.0001
		$E_{k\,(PUR)}$	43.9	6.9	6.4	0.000		Residual	9.0	122.1	13.6		
		R_D	6.7	1.5	4.5	0.001		Total	11.0	4539.9	412.7		
	8.0	P_E	12.5	3.1	4.0	0.005	0.723	Regression	2.0	382.1	191.1	9.1	0.011
		$E_{k\,(PUR)}$	4.7	15.8	0.3	0.775		Residual	7.0	146.6	20.9		
		R_D	5.2	2.0	2.6	0.038		Total	9.0	528.7	58.7		
8	6.0	P_E	62.6	2.4	26.1	<0.0001	0.992	Regression	2.0	5457.9	2729.0	458.5	<0.0001
		$E_{k\,(PUR)}$	68.6	7.2	9.5	<0.0001		Residual	7.0	41.7	6.0		
		R_D	5.0	1.1	4.7	0.002		Total	9.0	5499.6	611.1		
	7.0	P_E	44.9	4.0	11.3	<0.0001	0.952	Regression	2.0	3681.4	1840.7	89.4	<0.0001
		$E_{k\,(PUR)}$	57.1	13.3	4.3	0.002		Residual	9.0	185.4	20.6		
		R_D	4.9	1.8	2.7	0.025		Total	11.0	3866.8	351.5		
	8.0	P_E	20.3	2.4	8.4	<0.0001	0.922	Regression	2.0	846.2	423.1	41.5	0.000
		$E_{k\,(PUR)}$	17.4	7.1	2.4	0.045		Residual	7.0	71.3	10.2		
		R_D	7.8	1.4	5.5	0.001		Total	9.0	917.5	101.9		

Table S3. Results of non-linear regression analysis for electron transfer rate versus E_{PUR} curves. ETR_{max}: Maximum electron transfer rate (μmol electrons s^{-1} m^{-2}), α_{max}: efficiency of electron transport rate (μmol electron μmol^{-1} absorbed photon).

Model parameters								Analysis of Variance (Corrected for the mean of the observations)					
Growth pH	Measurement pH		Coefficient	SE	t	*p*	r^2		DF	SS	MS	F	*p*
6	6.0	ETR_{max}	35.3	0.4	87.0	<0.0001	0.999	Regression	1.0	1871.0	1871.0	14383.3	<0.0001
		α_{max}	0.4	0.0	59.9	<0.0001		Residual	12.0	1.6	0.1		
								Total	13.0	1872.6	144.0		
	7.0	ETR_{max}	41.0	3.3	12.6	<0.0001	0.963	Regression	1.0	2304.4	2304.4	258.7	<0.0001
		α_{max}	0.5	0.1	7.3	<0.0001		Residual	10.0	89.1	8.9		
								Total	11.0	2393.4	217.6		
	8.0	ETR_{max}	22.4	0.5	43.9	<0.0001	0.996	Regression	1.0	811.9	811.9	1857.1	<0.0001
		α_{max}	0.5	0.0	17.9	<0.0001		Residual	8.0	3.5	0.4		
								Total	9.0	815.4	90.6		
7	6.0	ETR_{max}	93.1	2.6	35.8	<0.0001	0.999	Regression	1.0	6602.7	6602.7	10308.1	<0.0001
		α_{max}	0.4	0.0	45.9	<0.0001		Residual	12.0	7.7	0.6		
								Total	13.0	6610.4	508.5		

Table S3. *continued*

Growth pH	Measurement pH		Coefficient	SE	t	*p*	r^2		DF	SS	MS	F	*p*
7	7.0	ETR_{max}	68.3	4.2	16.1	<0.0001	0.987	Regression	1.0	4969.9	4969.9	781.7	<0.0001
		α_{max}	0.5	0.0	11.9	<0.0001		Residual	10.0	63.6	6.4		
								Total	11.0	5033.5	457.6		
	8.0	ETR_{max}	32.4	1.1	28.2	<0.0001	0.992	Regression	1.0	1455.1	1455.1	950.5	<0.0001
		α_{max}	0.4	0.0	12.6	<0.0001		Residual	8.0	12.2	1.5		
								Total	9.0	1467.3	163.0		
8	6.0	ETR_{max}	58.4	5.8	10.0	<0.0001	0.970	Regression	1.0	3810.3	3810.3	261.3	<0.0001
		α_{max}	0.5	0.1	6.3	0.000		Residual	8.0	116.7	14.6		
								Total	9.0	3927.0	436.3		
	7.0	ETR_{max}	82.2	5.5	14.8	<0.0001	0.992	Regression	1.0	6334.7	6334.7	1175.7	<0.0001
		α_{max}	0.5	0.0	14.6	<0.0001		Residual	10.0	53.9	5.4		
								Total	11.0	6388.6	580.8		
	8.0	ETR_{max}	22.8	0.7	34.8	<0.0001	0.993	Regression	1.0	839.6	839.6	1162.5	<0.0001
		α_{max}	0.5	0.0	14.1	<0.0001		Residual	8.0	5.8	0.7		
								Total	9.0	845.4	93.9		
